# Amitozyn Impairs Chromosome Segregation and Induces Apoptosis via Mitotic Checkpoint Activation

**DOI:** 10.1371/journal.pone.0057461

**Published:** 2013-03-07

**Authors:** Bastien Herman, Aldrian Gudrun, Anatoly I. Potopalsky, Jadwiga Chroboczek, Sergey O. Tcherniuk

**Affiliations:** 1 Institut de Biologie Structurale, Centre National de la Recherche Scientifique (CNRS), Grenoble, France; 2 Centre de Recherche de Biochimie Macromoléculaire, Centre National de la Recherche Scientifique (CNRS), Montpellier, France; 3 Institute of Molecular Biology and Genetics, National Academy of Sciences of Ukraine (NAN Ukraine), Kiev, Ukraine; 4 Institute of Biochemistry and Biophysics, Polish Academy of Sciences (PAN), Warsaw, Poland; 5 Thérapeutique Recombinante Expérimentale/Techniques de l’Ingénierie Médicale et de la Complexité/Informatique, Mathématiques et Applications de Grenoble (Therex/TIMC/IMAG), Centre National de la Recherche Scientifique (CNRS)/Université Joseph Fourier (UJF), Domaine de la Merci, La Tronche, France; 6 Department of Biological Sciences, Academy of Young Scientists of Ukraine (AYSU), Kiev, Ukraine; University of Virginia, United States of America

## Abstract

Amitozyn (Am) is a semi-synthetic drug produced by the alkylation of major celandine (*Chelidonium majus L*.) alkaloids with the organophosphorous compound N,N’N’-triethylenethiophosphoramide (ThioTEPA). We show here that the treatment of living cells with Am reversibly perturbs the microtubule cytoskeleton, provoking a dose-dependent cell arrest in the M phase. Am changed the dynamics of tubulin polymerization *in vitro*, promoted the appearance of aberrant mitotic phenotypes in HeLa cells and induced apoptosis by the activation of caspase-9, caspase-3 and PARP, without inducing DNA breaks. Am treatment of HeLa cells induced changes in the phosphorylation of the growth suppressor pRb that coincided with maximum mitotic index. The dose-dependent and reversible anti-proliferative effect of Am was observed in several transformed cell lines. Importantly, the drug was also efficient against multidrug-resistant, paclitaxel-resistant or p53-deficient cells. Our results thus open the way to further pre-clinical evaluation of Am.

## Introduction

Together with surgery and radiotherapy, chemotherapy is one of the most effective tools for treatment of diverse neoplasms. Despite the huge arsenal of existing cytostatics, the development of new anticancer agents is necessary to overcome the rise in drug resistance. More than half of the known anti-proliferative drugs, such as Vinca alkaloids, taxanes or etoposide, are natural compounds or their derivatives. Natural compounds are employed in traditional and non-traditional medicine and present considerable advantages such as simplicity and relatively low cost of isolation on an industrial scale.

The medicinal plant celandine (*Chelidonium majus L.)* was used for the treatment of various diseases, and in particular of tumor neoplasms [1]–[3]. Detailed research on celandine composition showed that its anti-proliferative effect was due to the major extractable alkaloids: chelidonine, chelerythrine, sanguinarine, berberine and coptisine [4]. Despite their structural similarity, these compounds affect the living cells through different mechanisms. Chelidonine provokes mitotic arrest [5] and blocks the exit of dividing cells from anaphase. It is known to be able to modulate tyrosine kinase activity [6]. The proposed mechanism of chelidonine action, similar to that of colchicines, consists of inhibition of tubulin polymerization [7], [8]. Both sanguinarine and chelerytrine induce apoptosis in cancer cells [9], [10]. In addition, they exert a dose-dependent inhibition of angiotensin- and endothelin receptors [11] and inhibit the activity of some enzymes, such as lipoxygenases and aromatic amino acid decarboxylases [12], [13]. Sanguinarine has been shown to perturb microtubule assembly [14] and inhibit the activity of some enzymes [15], [16], while the mechanism of chelerythrine activity is not clear. It was proposed to be a potent inhibitor of protein kinase C [17], but this has later been questioned [18]. Sanguinarine, berberine and chelerythrine are powerful DNA intercalators; their activity, which provokes the double-strand breaks in DNA molecules, changes the physical properties of DNA and perturbs DNA replication and synthesis of mRNA [19]–[21]. Another celandine alkaloid, coptisine, decreases proliferation of vascular smooth muscle cells [22] and exhibits cytotoxicity against HT29, LoVo and L-1210 cells [23]. It is able to inhibit porcine pancreatic elastase and human sputum elastase [24]. However, coptisine has not been well studied and its mechanism of action remains unclear.

**Figure 1:**
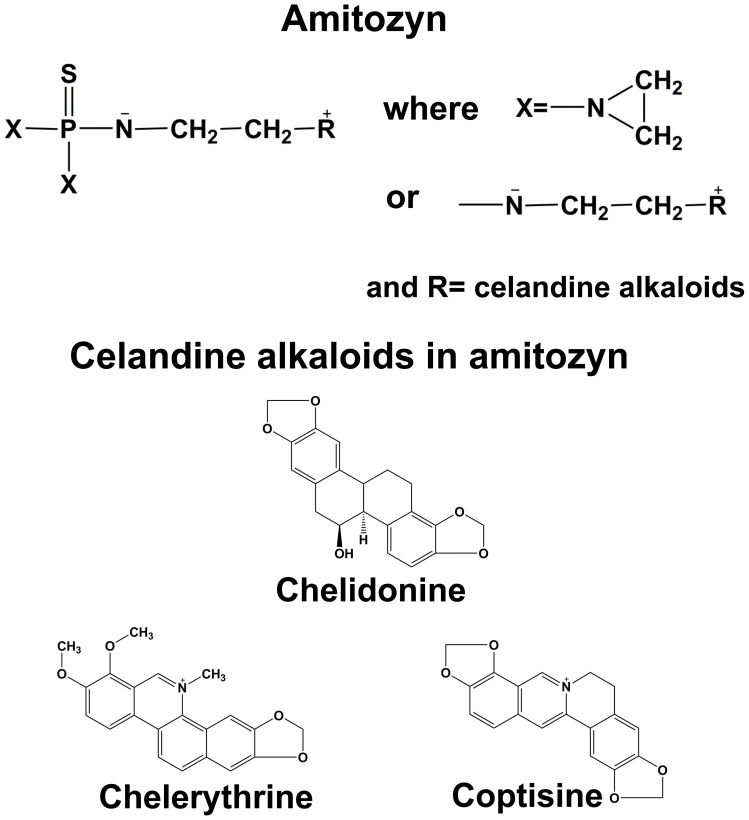
Structure of amitozyn and celandine alkaloids.

To enhance the antitumor activity and decrease the nonspecific cytotoxicity of celandine alkaloids it was proposed to modify them by alkylation. The alkylated pharmacological form called amitozyn (Am) is the result of alkylation of a mixture of celandine alkaloids (devoid of berberine) with N,N’N’-triethylenethiophosphoramide (ThioTEPA) (Figure 1). Am is widely used in folk medicine in Eastern Europe. Indeed, its anti-tumor potential has been demonstrated *in vitro* and *in vivo* in several tumor models [25]. However, the molecular mechanism of Am activity is not understood. In this work, we set out to elucidate its cellular effects. We found that Am accelerates the tubulin polymerization *in vitro* and promotes the appearance of aberrant mitotic phenotypes in HeLa cells. Am treatment provokes the mitotic block and induces apoptosis via mitotic checkpoint activation. Furthermore, Am inhibits the proliferation of transformed cell lines. Importantly, the drug is also efficient against multidrug-resistant, paclitaxel-resistant or p53-deficient cells.

## Materials and Methods

### Materials

The semi-synthetic drug Am was prepared as described in Supporting information at a concentration of 25 mg/ml. This preparation contains major celandine alkaloids (Figure 1, Figure S1, Table S1). Paclitaxel, etoposide, roscovitine, propidium iodide RNAse A and McCoy’s 5A medium were purchased from Sigma. AZ 3146 was purchased from Tocris Bioscience. Low melting agarose, SYBR Green I, advanced RPMI Medium 1640, D-MEM and fetal bovine serum were purchased from Invitrogen. LDH cytotoxicity kit was from Clontech. The following polyclonal rabbit Abs were used: anti-γ-H2AX and anti–phospho histone H3 from Upstate Biotechnology, anti-Pan-actin, anti-cleaved Caspase-9, anti-Caspase-3 and anti-poly ADP ribose polymerase (PARP), anti-phospho-PP1α (Thr320), rabbit anti-phospho-pRb (Ser780) from Cell Signaling, anti-BubR1 from Santa Cruz Biotechnology, FITC–conjugated donkey anti-mouse, anti-rabbit antibodies from Jackson ImmunoResearch and goat anti-rabbit and anti-mouse HRP-conjugated antibodies from Promega. The following mouse monoclonal Abs were used: anti-MPM-2 from Upstate Biotechnology, anti-β-tubulin from Sigma, anti-cyclin B1 from Santa Cruz Biotechnology, anti-pRb 4H1 from Cell Signaling, anti-p27 from Transduction Laboratories and anti-Bcl-2 from Dako.

The human HeLa, KB3, HT29, HCT116, A549, MESSA and murine B16 and GL26 cell lines were purchased from ATCC. The HeLa cells stably expressing histone 2B fused to eGFP (HeLa-H2B-eGFP) were kindly provided by N. Morin (CRBM, Montpellier, France). HCT116 p53(^−/−^) with homozygous knock-out of p53 were kindly provided by D. Skoufias (IBS, Grenoble, France). Taxol resistant A549T12 cells were obtained with permission from S. Horwitz (Albert Einstein College of Medicine, New York, NY, USA). Taxol resistant KB-15-PTX/099 cells were kindly provided by S. Loganzo (Wyeth Research, Pearl River, NY, USA). The MESSA Dx5 cells were kindly provided by L. Lafanechère (CNRS, UMR 5168/CEA/IRTSV, Grenoble, France).

### Flow Cytometry

Cells at 60–70% of confluence were treated with 0 to 500 µg/ml Am for 3, 6, 9, 12, 24, 36, 48, 60 and 72 h and subsequently collected by pooling together the non-attached and attached cells. Then cells were analysed by two-dimensional flow cytometry as described previously [26]. Briefly, cells following fixation in ice cold 90% methanol, were washed three times in PBS and then incubated with anti-MPM2 MAb diluted 1∶500. Then cells were washed twice as described above and subsequently incubated for 30–40 min at 37°C with FITC-conjugated anti-mouse Ab diluted 1∶250. After two washing steps, the cells were labeled with propidium iodide (PI), a DNA marker. The suspension of stained cells was analyzed by FACScan (Becton Dickinson flow cytometer), using CellQuest software. For each sample 10 000 events were collected and the aggregated cells were gated out.

### Immunoblotting

Am-treated and control HeLa cells were harvested, washed with PBS and subsequently lysed in lysis buffer (50 mM Tris, pH 7.4, containing 250 mM NaCl, 0.1% NP-40, 0.1 mM PMSF, aprotinine at 10 µg/ml, leupeptine at 10 µg/ml and 100 mM NaF) for 30 min on ice. Then, the cell lysate was centrifuged for 10 min at 13 000 rpm and the concentration of soluble proteins in supernatant was measured by the Bradford method. Equal portions of protein (20 µg) were resolved by SDS/PAGE, electrotransferred onto the nitrocellulose membrane and treated with the appropriate primary Ab diluted 1∶1000. For loading controls the anti-pan-actin and anti-β-tubulin antibodies were used at 1∶1000 dilution. After overnight incubation with Ab, the membrane was quickly washed and incubated for 1 h at room temperature with the secondary HRP-conjugated anti-rabbit or anti-mouse antibody, diluted at 1∶5000. Finally, the membranes were developed with ECL (ThermoScientific).

### Cell Culture, Immunofluorescent Microscopy and Live Cell Imaging

The antiproliferative effect of Am was analyzed on 13 different cell lines. HeLa, Hela cells stably expressing histone 2B fused to eGFP (HeLa-H2B-eGFP), GL26 and B16 cells were grown in the DMEM medium supplemented with 2 mM L-glutamine, 1% penicilin/streptomycin and 10% FBS. KB and KB-15-PTX/099 cells were grown in the same medium supplemented with 20% FBS. The KB-15-PTX/099 cells were grown in the presence of 2 nM paclitaxel. MESSA, MESSA (Dx5) MDR, A549 and A549T12 were cultivated in the RPMI 1640 medium supplemented with 2 mM L-glutamine, 1% penicilin/streptomycin and 10% FBS. The medium for A549T12 cells was additionally supplemented with 2 nM paclitaxel. HCT116, HCT116 p53(^−/−^) and HT29 were cultivated in McCoy’s 5A medium supplemented with 1% penicillin/streptomycin and 10% FBS. All cell lines were maintained in a humid incubator at 37°C in 5% CO_2_.

Cells were left to adhere for at least 24–36 h onto poly-D-lysine–coated coverslips placed in 60 cm^2^ Petri dishes before drug addition. When cells reached the 60–70% confluence, the medium was replaced with a fresh one supplemented with Am at concentrations up to 500 µg/ml. After appropriate time of exposure to Am, cells were fixed by immersing the coverslips with attached cells in 2% paraformaldehyde in PBS for 20 min at 37°C. After three PBS washes, cells were permeabilized with 0.2% Triton in PBS for 5 min at room temperature and immediately washed three times with PBS, each time for 5–10 min. Cells were treated with anti-β-tubulin and anti-γ-H2AX Abs diluted 1∶400 and 1∶100, respectively, in the antibody buffer (PBS containing 3% BSA, 0.05% Tween and 0.02% sodium azide). Cells were subsequently stained with a FITC-conjugated donkey anti-mouse and anti-rabbit secondary Abs at 1∶250 dilution for 30 min at 37°C. Finally cells were counterstained with PI at 0.5 µg/ml. Images were captured with a BX61 motorized research microscope (Olympus) and analyzed using Volocity software (Improvision).

To visualize the dynamics of cell growth in the presence of Am, time-lapse microscopy was performed. For imaging we used HeLa cells synchronized by double thymidine block (Supporting information) and non-synchronized HeLa cells stably expressing histone 2B fused to eGFP. Cells were filmed with a Leica DMIRE2 inverted microscope as described previously [27].

### Checkpoint Inhibition Experiments

To analyse whether Am induced mitotic checkpoint requires the Cdk kinase activity, the chemical inhibitor roscovitine [28] was used. After double thymidine block, performed as described in Supporting information, the HeLa cells were released in drug-free medium. After 6 h the cells were treated with Am (250 µg/ml) and released 11 h later under four different conditions: 250 µg/ml Am, 250 µg/ml Αm with 10 µΜ roscovitine, 250 µg/ml Αm with 4 µΜ inhibitor of TTK1 kinase AZ 3146 [29], and in the drug-free medium. The time of mitotic exit and presence of a mitotic checkpoint were analysed by time-lapse microscopy and western blot, respectively, as described above.

### Microtubules Polymerization Assay

Effect of Am on tubulin polymerization was studied with tubulin from bovine brain, prepared as described [30]. Tubulin polymerization was carried out at 37°C in PEM buffer (100 mM PIPES, 1 mM Na-EGTA, and 1 mM MgCl_2_, pH 6.9) by mixing 60 µM of tubulin, 4% DMSO and different Am concentrations, in total volume 50 µl. Controls were carried out in the presence of 2 µM paclitaxel or nocodazole at 20 µg/ml. All measurements were performed with 96-well Sunrise photometer (Tecan, Maennedorf, Switzerland) at 340 nm with intervals of 7 sec. Statistical significance of data was analyzed by Microsoft Excel.

To study Am effects on total polymer mass of tubulin, tubulin polymerization was carried out for 24 h at 37°C either in the presence of different Am amounts, or water/polyethylenglycol 400/dymethylsulfoxide in proportions 1.5/1.5/2 (polymerization control), or with paclitaxel (stabilization control), or nocodazole (depolymerization control). The incubation mixtures were centrifuged at 50 000 rpm for 30 min, and the pellet and the supernatant fractions were analyzed by SDS-PAGE.

### DNA Comet Assay

Comet assay was used to determine the ability of Am to induce double-strand breaks in HeLa DNA. Cells grown in 24-well plates to 60–70% confluence were treated with different concentrations of Am for 12 h, scraped mechanically, suspended in medium and centrifuged for 5 min at 1000 rpm. Recovered cell pellet was suspended in 1 ml PBS (pH 7.4), and 10 µl of cell suspension was mixed with 90 µl of 0.5% low-melting agarose diluted in PBS and preheated to 37°C. The cell-agarose mixture was applied onto microscopic slides. After agarose solidification the slides were placed in the cold (4°C) lysis buffer (10 mM Tris, pH 10, 2.5 M NaCl, 100 mM EDTA, 1% Triton) for 1 hour, then treated with alkaline solution (300 mM NaOH, 1 mM EDTA, pH 13) for 20 min. The slides were washed twice in TBE buffer, each time for 10 min. Next, the slides were placed in TBE buffer and the electrophoresis was carried out during 10–15 minutes at 1 V per 1 cm of distance between electrodes. Then the slides were washed twice with distilled water (2×5 min) and placed in 70% ethanol for 5–10 min, dried on air, stained with SYBR Green I and observed in fluorescent microscope at 480 nm. The comet tails of Am-treated cells were compared with comet tails of untreated (negative control) cells or treated with 5 µg/ml etoposide (positive control).

### Cell Viability and Cytotoxicity Assay

Portions of 1–2×10^4^ cells/well were seeded in 24-well plates and incubated with 0 to 500 µg/ml of Am for 24 to 72 h. At the desired time point the medium was removed and tested in triplicates using the LDH cytotoxicity kit. The cytotoxicity assay was performed and results were calculated as described in the kit manual. In parallel, the cells were harvested, treated with 0.2% trypan blue and counted. The cell viability was estimated as percentage of live cells compared to control. The viability assay was performed in duplicate in three independent experiments. The statistical significance of the difference between the control and treated groups was determined by *t*-criterion of Student. P-value ≤0.05 was considered to be statistically significant.

## Results

### Qualitative and Quantitative Analysis of Am Composition

Liquid chromatography-electrospray ionization-mass spectrometry (LC-ESI-MS) and LC-ESI-MS/MS experiments were carried out to determine Am composition. Peak area % comparison was used to identify the relative contents of alkaloids in Am (Table S1, Figure S1). The identity of the main alkaloid chelidonine was confirmed by detection of molecules with the same MS/MS- fragmentation pattern as chelidonine standard (Figure S2A, B, C). Chelidonine appears at two different retention times (t_R_) which can be due either to the presence of two isomeric forms or different counterions. The results of MS analysis suggest that ThioTepa is not covalently linked to alkaloids but seems to dimerize and probably forms complexes with alkaloids by electrostatic interactions.

A standard HPLC method with UV detection at 280 nm was used for the quantitative determination of the main alkaloid chelidonine in amitozyn. The chelidonine standard concentration was 1.25 mM. Comparison of peak areas from integration of the detector signals during elution showed a concentration of 443 µM of chelidonine in the Am preparation. The concentrations of chelerythrine (30 µM), coptisine (79 µM) and ThioTEPA dimer (56 µM) were calculated according to the relative peak areas.

### General Effect of Am on HeLa Cells

**Figure 2:**
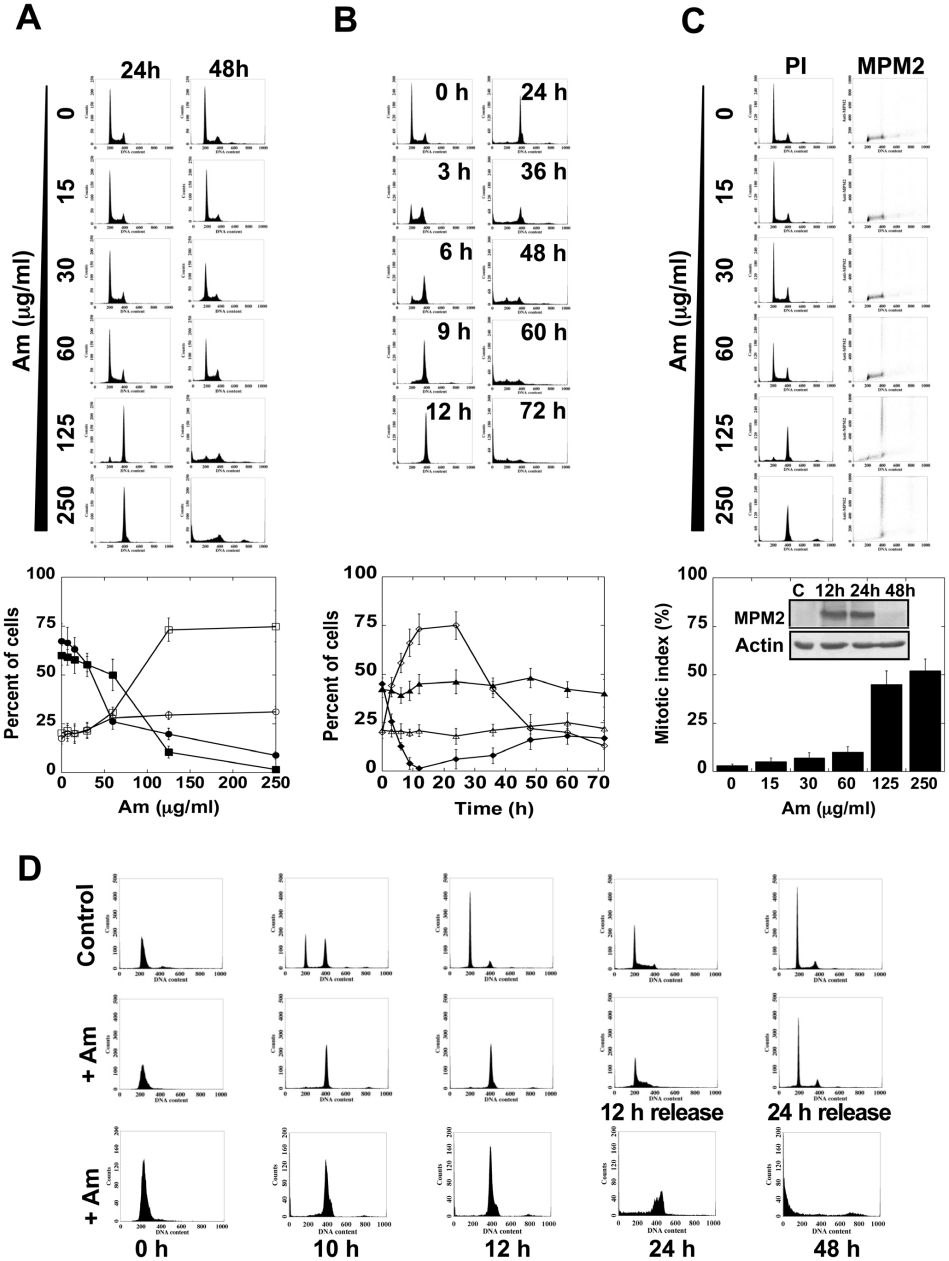
General characterization of amitozyn effect on HeLa cells. (A) Cells were exposed to different concentrations of Am for 24 and 48 h and the percent of 2N and 4N cells in three independent experiments was estimated by FACScan and plotted in graph shown in the lower panel. ▪- percent of 2N cells after 24 h treatment; □ - percent of 4N cells after 24 h treatment; • - percent of 2N cells after 48 h treatment; 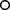 - percent of 4N cells after 48 h treatment. (B) Kinetics of Am effect on HeLa cells. Cells were exposed to 125 µg/ml of Am for periods from 0 to 72 h and analyzed by FACScan. The percent of 2N and 4N cells from three independent experiments was plotted in graph shown at the bottom. ▴ - 2N cells in control; Δ- 4N cells in control; ♦ - 2N cells after treatment with Am; ◊ - 4N cells after treatment with Am. (C) FACScan analysis of mitotic cell ratio after Am treatment. The HeLa cells were exposed for 24 h to different Am concentrations, fixed with methanol, stained with PI and MPM2-FITC and analyzed by FACScan (upper panel). The average mitotic index of three independent experiments was plotted in the graph (lower panel). The Western blot inset shows the MPM2 level at indicated time points. (D) Reversibility of Am effect. The HeLa cells were synchronized in the G1/S phase by double thymidine block and released by medium addition (top row), exposed to Am (250 µg/ml) for 12 h with subsequent release in medium for 12 and 24 h (second row) or released in Am (250 µg/ml) during the entire experiment (last row). Cells were analyzed by FACScan as described in Materials and methods.

Treatment of non-synchronized cells with Am at concentration of 125–500 µg/ml led to cell arrest in the G2/M phase. The percentage of 4N cells depended on the Am concentration and exposure time (Figure 2A, B). The lowest Am concentration that showed nearly the highest arresting effect was 125 µg/ml (Figure 2A). Exposure to 125 µg/ml Am for 3 h increased the percent of 4N cells to 44±4%. This number increased to 73±7% or 75±6% when exposure was prolonged to 12 or 24 h (Figure 2B). After a 24 h exposure, the number of 4N cells decreased, with concomitant increase in the number of apoptotic cells, revealed by an increase of the sub-2N-peak (Figure 2B). Staining with mitotic protein monoclonal 2 (MPM2) antibody that recognizes a family of proteins sharing a common mitotic cells phosphorylated epitope [31] demonstrated, that treatment with 250 µg/ml Am for 24 h resulted in an increase of mitotic index from 2±0.7% for untreated cells to 52±6% (Figure 2C). The highest level of MPM2 protein was observed after 12–24 h treatment with Am, whereas with a longer Am treatment (48 h) the level of MPM2 significantly decreased (Figure 2C, WB). These results together show that amitozyn arrests HeLa cells in M phase in the concentration- and time-dependent manner.

### The Effect of Am is Reversible

To see if the effect of Am was reversible, HeLa cells were synchronized in the G1/S phase by double thymidine block and released either by addition of medium or in the presence of 250 µg/ml Am (Figure 2D). Cells, which after a 12 h treatment with 250 µg/ml Am were arrested in metaphase, upon medium release were able to overcome mitotic arrest and underwent mitosis and cell division (Figure 2D, middle row). However, when cells were treated with Am for 24 h, they were unable to overcome mitotic arrest and, consequently, underwent apoptosis or became polyploid (Figure 2D, third row). These results indicate that cells blocked in mitosis after Am treatment did not undergo apoptosis immediately and demonstrate that Am is a reversible drug.

### Am Induces Apoptosis in HeLa Cells and Modulates the Level of Growth Suppressor pRb

**Figure 3:**
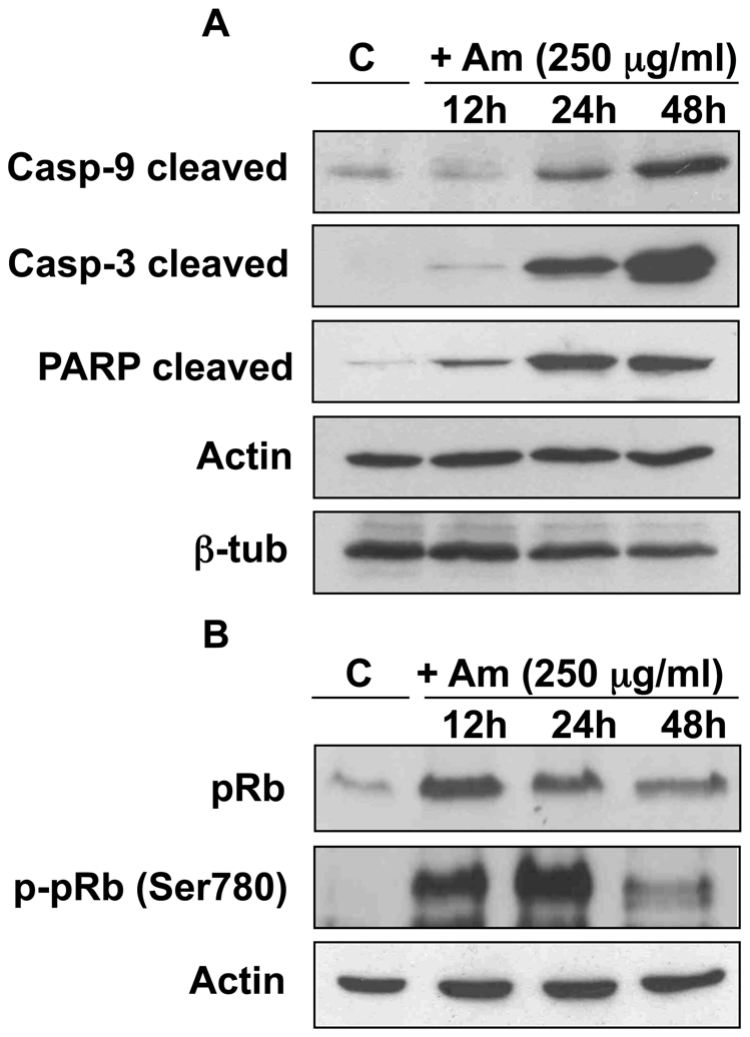
Activation of apoptotic markers and modulation of pRb level upon Am application. HeLa cells were treated with 250 µg/ml Am for 12, 24 and 48 h. As a control (C) we used untreated cells grown for 48 h. Cell lysates were prepared as described in Materials and methods, and the level of activated caspase-9, caspase-3 and cleaved PARP (A), and total pRb and pRb phosphorylated at Ser 780 (B) was analyzed by Western blot. β-tubulin and actin were used as loading controls.

We estimated the level of common effectors of apoptosis: activated caspase-9, caspase-3 and poly (ADP-ribose) PARP-1 [32]–[34] and growth suppressor pRb. Treatment of HeLa cells with 250 µg/ml Am increased the quantity of activated caspase-9, caspase-3 and PARP-1 (Figure 3A), in agreement with FACScan data that showed mitotic arrest upon a 12 h Am treatment (Figure 2A). In parallel, the Am treatment upregulated the level of pRb protein and its inactive forms, phosphorylated at Ser 780 (Figure 3B), which coincided with mitotic events and induction of apoptosis.

### Am Perturbs the Cell Cycle in HeLa Cells and Induces Mitotic Checkpoint

**Figure 4:**
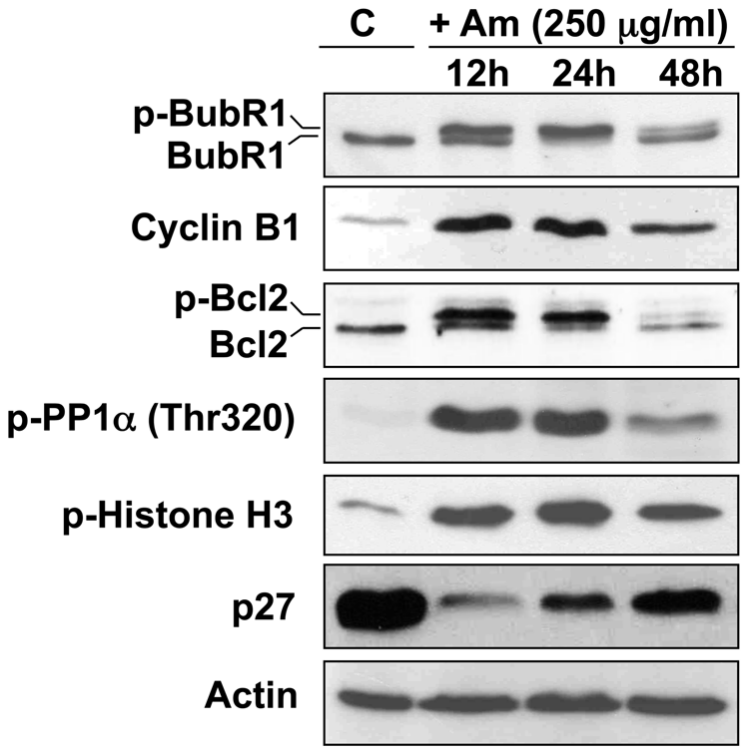
Perturbation of cell signalling and mitotic checkpoint activation in HeLa cells upon Am treatment. HeLa cells were treated with 250 µg/ml Am for 12, 24 and 48 h. As a control (C) we used untreated cells grown for 48 h. Cell lysates were prepared as described in Materials and methods, and the level of indicated proteins was analyzed by Western blot. Actin was used as loading control.

Phosphorylated BuBR1 is an indicator of mitotic checkpoint [35]. An increase in the phosphorylation of BuBR1 was observed upon Am treatment (Figure 4). A similar timing was associated with modulation of the amount of cyclin B1 upon Am treatment. Cyclin B1 amount was at first elevated, and decreased somewhat at 48 h treatment (Figure 4). Moreover, Bcl2 protein, known as a marker of M-phase events [36], was phosphorylated upon Am treatment, which suggests the appearance of mitosis-arrested cells [37]. Furthermore, Am treatment for 12–24 h significantly increased the level of phospho-PP1α (Thr 320), suggesting PP1 inhibition [38]. Such multiple phosphorylation events that occur predominantly during the early and mid-mitosis, have been observed in many cell types arrested at mitosis [39]. The sustained mitotic status of HeLa cells exposed to Am was supported also by phosphorylation of the histone H3 that is not observed in randomly cycling cells [40] (Figure 4). The mitotic block observed upon Am treatment is thus most probably due to activation of the mitotic spindle assembly checkpoint.

**Figure 5:**
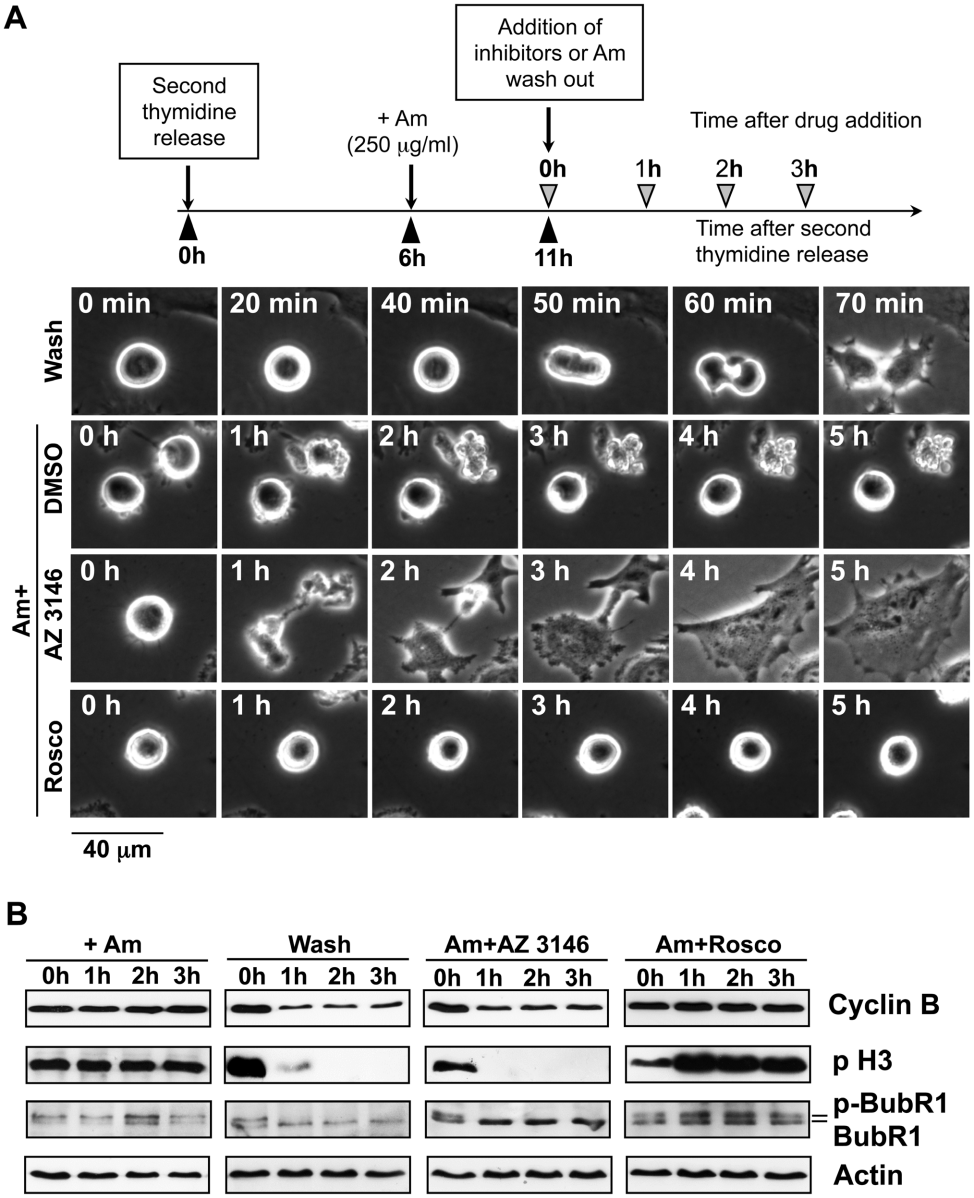
Effect of roscovitine and AZ 3146 on the spindle checkpoint in HeLa cells blocked by Am. Time lapse (A) and western blot analysis (B) analysis of mitotic cells progression after treatment with roscovitine and AZ 3146. HeLa cells were synchronized by double thymidine block and released in the drug free medium. After 6 h cells were treated with 250 µg/ml Am. 5 h later the DMSO (control), roscovitine (10 µM) and AZ 3146 (4 µM) were added. Another cell portion was released in a drug-free medium (wash panel). At indicated timepoints cells were trypsinized and cell lysate was analysed by western blot as described in Materials and methods. Actin was used as loading control.

Am treatment affected also the levels of negative regulators of cell cycle such as p27. At 12 h Am did not arrest cell cycle progression in the G1 phase, which can be judged by p27 extinction. However, a slight p27 increase at 24 h and 48 h suggests that the appearance of tetraploid G1-phase cells arose from 4N cells blocked in mitosis, which is in agreement with the time-lapse data (Figure S3A, B). Some Am-treated cells exited from mitosis forming the micronucleated cells and entered the next G1 phase with subsequent apoptosis (Figure S3A). Interestingly, the treatment of mitotic HeLa cells with checkpoint inhibitor AZ 3146 (but not with roscovitine) decreased the level of cyclin B, histone H3 phosphorylated at Ser 10 and a phosphorylated form of BubR1 that overrode the mitotic checkpoint and promoted the mitotic exit even in the presence of Am (Figure 5A, B). Taken together, these results show that Am synchronizes HeLa cells in M phase by mitotic block, activates the mitotic checkpoint leading to cell death in mitosis (9±2% of cells) or to the appearance of tetraploid micronucleated cells (91±2% of cells) (Figure S3B), with ensuing apoptosis and death. Furthermore, the TTK1 inhibitor AZ 3146 (but not roscovitine) could override the Am induced mitotic checkpoint accelerating the mitotic exit (Figure 5A, B).

### Am induces an Aberrant Phenotype in HeLa Cells

**Figure 6:**
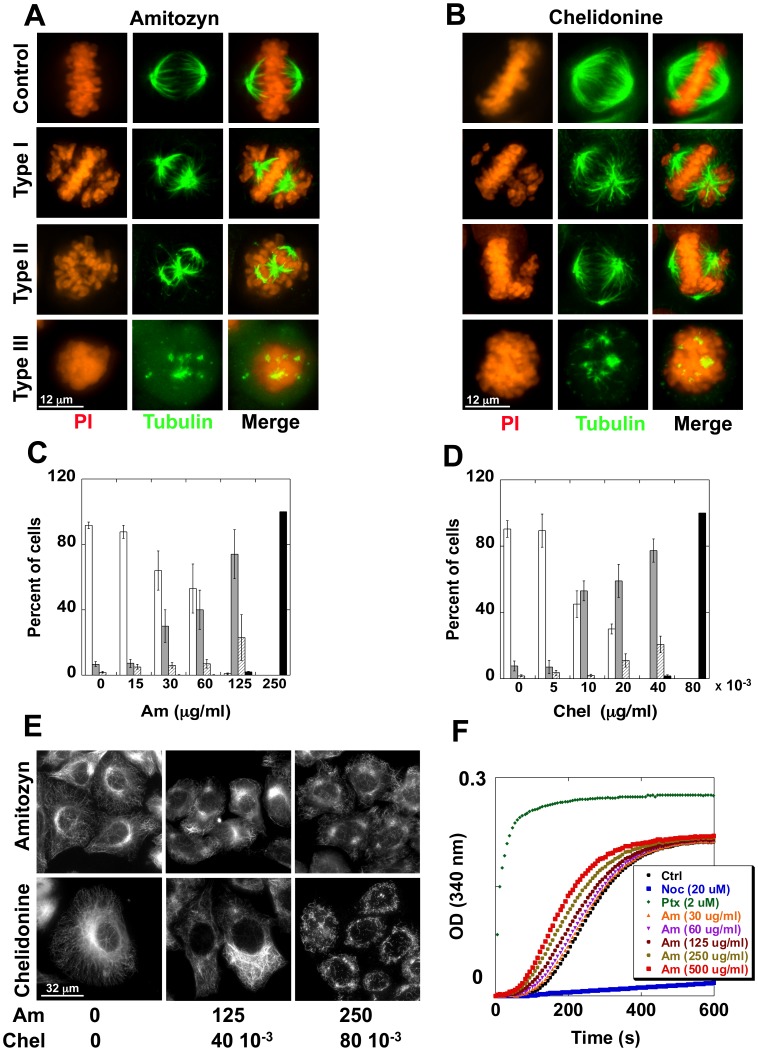
Effect of amitozyn on cell morphology and polymerization of tubulin *in vitro*. HeLa cells were treated with 0, 15, 30, 60, 125, 250 µg/ml amitozyn for 8 h, fixed and stained with anti-tubulin Ab (green) and PI (red). A and B, the mitotic phenotypes of HeLa cells observed after amitozyn and chelidonine (Chel) treatment, respectively. C and D, ratios of different mitotic phenotypes in cell population after Am and Chel treatment, respectively. Percent of cells with **white** - normal mitotic spindle; **grey** - abnormal mitotic spindle of type I; **shaded** - abnormal mitotic spindle of type II; **black** - abnormal mitotic spindle of type III. (E) Interphase cell observed in the presence of amitozyn and chelidonine. HeLa cells were exposed to amitozyn and chelidonine at indicated concentrations, fixed and stained with anti-tubulin Ab. Drug concentration is shown in µg/ml. (F) Effect of amitozyn on tubulin polymerization *in vitro*. Tubulin (60 µM) was polymerized for 10 min at 37°C in the presence of 0–500 µg/ml amitozyn as described in Materials and methods.

After exposure of HeLa cells to Am, we observed an increasing number of cells in mitosis. Detailed observation showed that the dose-dependent treatment with Am led to appearance of cells with the aberrant mitotic spindles. In cells treated with 30–125 µg/ml Am we observed bipolar and multipolar mitotic spindles (Figure 6A, second and third row, respectively). In cells with bipolar aberrant phenotype some chromosomes were not correctly aligned in the metaphase plate and were localized near the spindle pole. Furthermore, the distance between spindle pole was smaller (8.5±0.5 µM) than in untreated cells (14.9±0.9 µM) (Figure 6A, compare first and second row and Figure S4A). The multipolar aberrant cells had more than two poles and more than one metaphase plate. In contrast, upon treatment with high Am concentration (250–500 µg/ml), the mitotic spindle was completely destroyed, resulting in cells with multiple diffuse tubulin aggregates in the cytoplasm (Figure 6A, last row). The observed phenotypes were not similar to those induced by paclitaxel whereby two or more large poles, significantly bigger compared to tubulin aggregates, were observed after Am treatment, but were comparable to the phenotypes induced by chelidonine, vinblastine, nocodazole and colchicine (Figure 6B and Figure S4B), suggesting that Am might affect the microtubule dynamics by an analogous mechanism.

In cells treated with 15–30 µg/ml Am, the number of aberrant mitotic cells of type I increased about 5–8 fold compared to control, while treatment with 125 µg/ml Am led to disappearance of cells with normal mitotic spindles, with concomitant induction of about 75% aberrant bipolar mitotic cells of type I and about 25% of aberrant multipolar cells of type II (Figure 6C). Am at 250 µg/ml resulted in appearance of mitotic cells of type III, all containing tubulin aggregates diffused in the cytoplasm. The interphase cells treated with 15–125 µg/ml Am had normal or weakly affected microtubules. When interphase cells were treated with 125–250 µg/ml Am, their microtubules appeared to change the shape and density (Figure 6E, first row), which was similar to images observed after treatment with 40–80×10^−3^ µg/ml chelidonine (Figure 6E, second row). As shown in Figure 2D, cells synchronized in the G1/S phase can enter mitosis in the presence of Am, without arrest in the phase G1, S or G2, suggesting that Am blocks the cells in M phase, similarly to the majority of microtubule drugs.

### Am Changes the Dynamics of Tubulin Polymerization *in vitro*

We analyzed the polymerization profile of bovine tubulin in the presence of Am, in comparison with effect exerted by paclitaxel and nocodazole. In the presence of 30–500 µg/ml Am the rate of tubulin polymerization increased. However, the total quantity of polymerized tubulin was not changed by Am (Figure 6F). In contrast to Am, chelidonine inhibited the tubulin polymerization in concordance with previously published data [7] (Figure S5). As the polymerization profile was different from the one obtained in the presence of nocodazole, chelidonine and paclitaxel, it can be inferred that Am has a different mechanism of action *in vitro*, and is most possibly acting by accelerating tubulin assembly.

### Am does not Induce dsDNA Breaks

**Figure 7:**
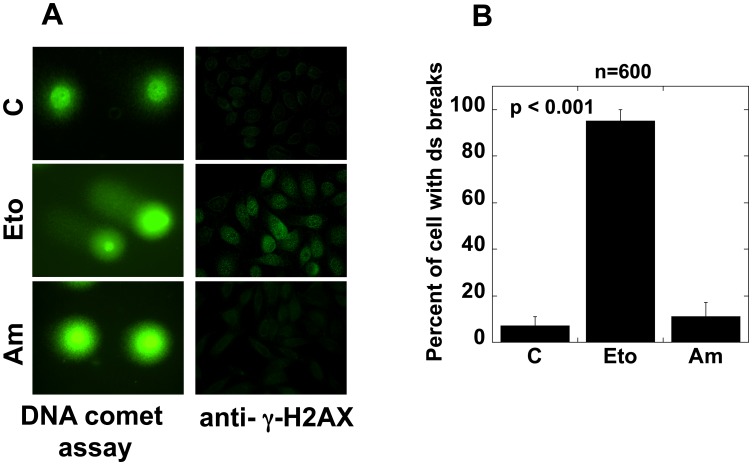
Effect of amitozyn on DNA integrity. (A) Double-strand breaks analyzed by DNA comet assay (left column) and by immunofluorescence microscopy (right column). Top row - untreated HeLa cells (C), second row – cells treated with 5 µg/ml etoposide (positive control), last row – cells treated with 250 µg/ml amitozyn for 12 h. The tail in the comet assay and histone γ-H2AX visualized by immunofluorescence microscopy shows the presence of DNA double-strand breaks. (B) Percentage of cells containing double-strand breaks revealed by DNA comet assay.

Some DNA damaging agents can provoke the arrest of mammalian cells during the G2/M transition. Since Am includes the modified chelerythrine that can potentialy induce the dsDNA breaks, we checked the potential of Am to damage DNA in HeLa cells. The appearance of dsDNA breaks appearing in the form of tails in the comet assay in Am-treated cells was investigated in comparison with etoposide-treated and untreated cells. We used etoposide for positive control because etoposide is a cancer drug that induces strand breaks in cellular DNA by inhibition of topoisomerase II-mediated religation of cleaved DNA molecules [41]. Treatment of HeLa cells with 500 µg/ml Am for 12 h did not increase the ratio of cells with dsDNA breaks, while a significantly higher number of tails was seen upon etoposide treatment (Figure 7A, B). To avoid the induction of dsDNA breaks caused by apoptosis, the treatment was not carried out beyond 12 h. The dsDNA breaks induced the phosphorylation of the H2AX histone that can be visualized by immunofluorescent staining of phosphorylated γ-H2AX. However, no γ-H2AX histone could be seen either in cells treated with 500 µg/ml Am for 12 h or in untreated ones (Figure 7A), thus confirming that Am does not induce dsDNA breaks. These results indicate that Am affects cells in a different way than DNA-damaging agents.

### Am Inhibits Cell Proliferation

**Figure 8:**
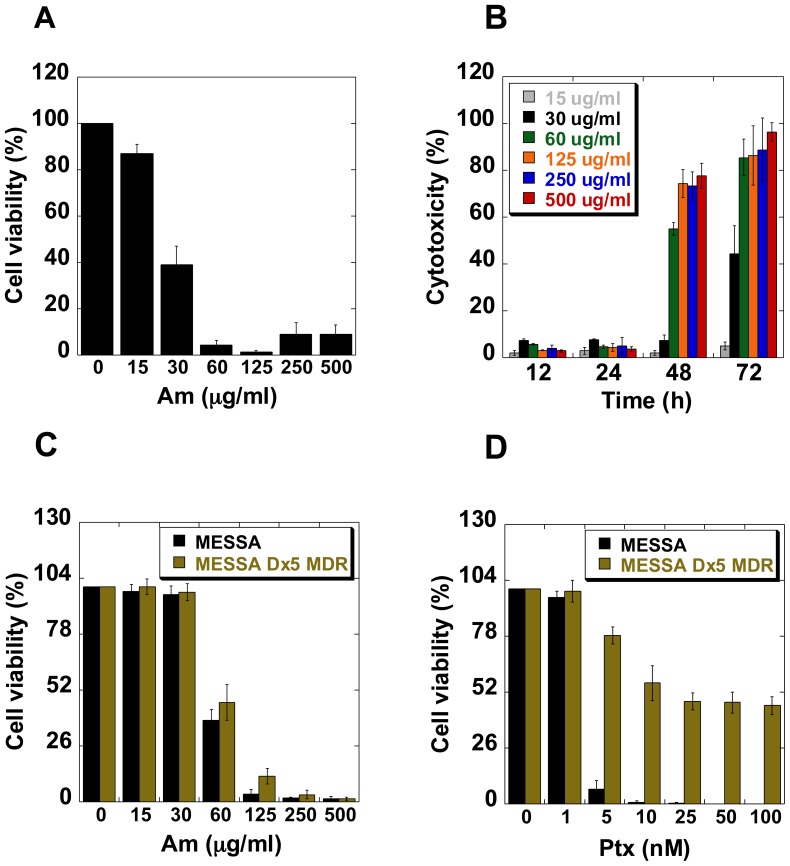
Effect of Am on cell viability. (A) HeLa cells were treated with Am for 72 h as described in Material and Methods and cell viability was measured by trypan blue exclusion. The cytotoxic effect (B) of Am was estimated after indicated periods using the LDH kit. Average data of three independent experiments are presented. (C) MESSA cells and multidrug resistant MESSA Dx5 cells were treated either with Am or with paclitaxel (D) for 72 h and cell viability was measured as described above.

We observed that Am decreased cell viability and induced cytotoxic effect in HeLa cells. Approximatly half of HeLa cells were unable to proliferate when 30 µg/ml of Am was applied for at least 72 hours. Cell proliferation was nearly totally blocked by Am above 60 µg/ml (Figure 8A). In parallel, Am cytotoxicity was estimated using the lactate dehydrogenase (LDH) release assay. Lactate dehydrogenase (LDH) is a soluble cytosolic enzyme that is released into the culture medium following loss of membrane integrity and which serves as a general assay to asses cytotoxicity resulting from the activity of chemical compounds or the enviromental toxic factors [42]. An Am treatment for 12–24 h induced a low cytotoxic effect (about 2–7%), but after 48 h of treatment the cytotoxicity increased up to 80–96% (Figure 8B). The time of maximum LDH release (48 h) coincided with sustained activation of caspase-3, caspase-9 and PARP (Figure 3A). These results, when combined with the observed phenotypic changes induced by Am, demonstrate that cell death might result from prolonged Am-induced mitotic arest or aberrant exit from mitosis leading to appearance of micronucleated cells and apoptosis.

**Figure 9:**
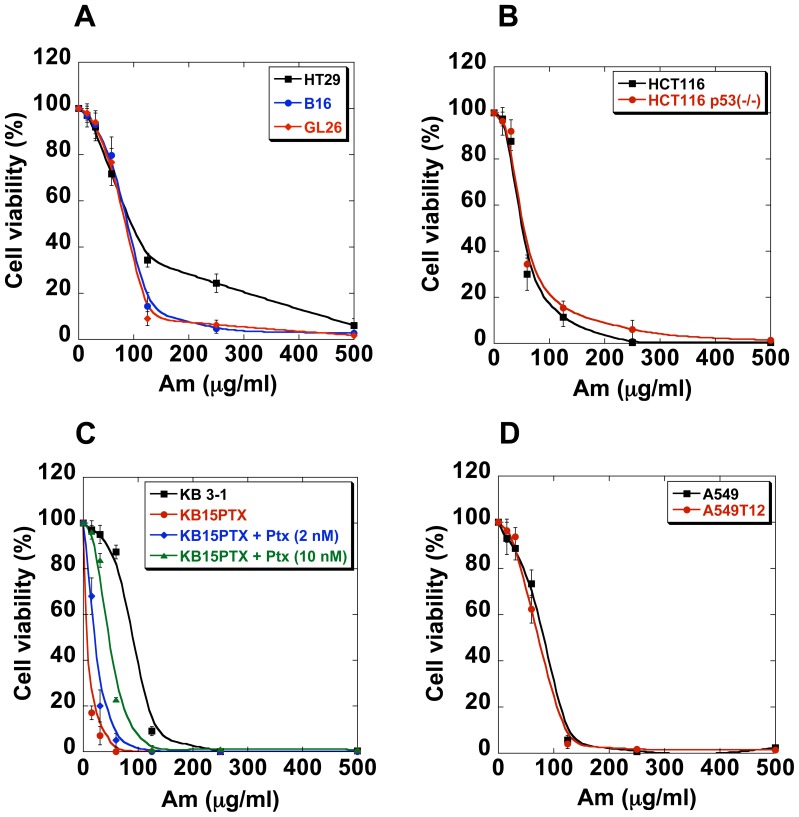
Anti-proliferative effect of amitozyn in different cell lines. Cells were treated with Am at indicated concentration for 72 h as described in Material and methods. Cells viability was determined by trypan blue exclusion.

Promising results obtained in the HeLa model encouraged us to test this drug on other cells, such as cells with chromosomal instability (CIN) and also cells with wild type p53 and devoid of p53, mutidrug-resistant or paclitaxel-resistant cells and their drug-sensitive counterparts. In addition, two mouse cancer lines were also analyzed. To be able to compare the Am effects we determined cell viability for different cell lines (Figure 8 and Figure 9) and calculated the IG_50_ for different periods of Am treatment (Table 1).

**Table 1:** Growth inhibition expressed in IG_50_ values of amitozyn for different cell lines.

Cell line	IG_50_ (µg/ml)
Hela	27±3
HT29	96±2
HCT116	54±3
HCT116 p53(−/−)	53±6
MESSA	57±9
MESSADx5 MDR	61±12
KB3-1	47±6
KB15PTX	6.9±2.4
KB15PTX+Ptx (2 nM)	18.6±3.2
KB15PTX+Ptx (10 nM)	45±4.1
A549	82±7
A549T12	77±3
B16	88±2.5
Gl26	83±3

Cells were exposed to 0–500 µg/ml Am. At 72 h cells were harvested by trypsinization and the ratio of viable cells was estimated by trypan blue exclusion. The IG_50_ value was estimated from cell viability plots and presents the average value of three independent experiments.

HT29, a cancer cell line with chromosomal instability have been shown to be unable to produce mitotic checkpoint regulator protein BUBR1, which results in an abnormal response to microtubule disrupting drugs, leading to a small amount of mitotic cells and no clear peak in mitotic index [43]. Despite the low doubling time of HT29 cells (19.5 h), the IG_50_ for 72 h of Am treatment were relatively high, (Table 1), however, at 120 h Am exposure, the IG_50_ decreased to 40±4 µg/ml (data not shown), showing that massive cell death was induced after prolonged Am treatment. These data suggest that even if Am-treated CIN cells can escape mitotic catastrophe through increase in ploidy, the accumulation of chromosomal aberrations becomes critical at a certain moment and provokes cell death. The precise mechanism of this phenomenon requires additional investigation.

It is known that many cancer cells express an inactivated p53 protein, which changes their sensitivity to drugs [44]–[47]. To find out if the effect of Am also depends on p53 status, we used the HCT116 and HCT116 p53(^−/−^) cell lines. We observed that the presence of intact p53 did not increase significantly the sensitivity of HCT116 to Am after 72 h of exposure (Table 1 and Figure 9).

The effect of Am and paclitaxel on MESSA multidrug-resistant (Dx5) and MESSA non-resistant cells was investigated. We observed that the IG_50_ for paclitaxel at time point 72 h was 2±0.2 nM for MESSA and 24±2 nM for MESSA Dx5 MDR cells (Figure 8C, D). Contrary to this, there was no significant difference between amitozyn IG_50_ for MESSA and MESSA Dx5 MDR cells at the same time point (Figure 8C, D and Table 1). This is a remarkable result in the context of chemotherapy for drug-resistant cancers since the MESSA Dx5 cells contain an active PgP efflux pump [48].

Since Am activated tubulin polymerization *in vitro*, it was of interest to study its effect on cells resistant to paclitaxel, a commonly used microtubule stabilizer. We analyzed the effect of Am on paclitaxel-resistant KB-15-PTX/099 and A549-T12 cells and its paclitaxel-sensitive counterparts, KB and A549, respectively [49], [60]. Paclitaxel-resistant KB-15-PTX/099 that were grown in the paclitaxel-free medium during 72 h, were about 12 times more sensitive to Am than their paclitaxel-sensitive analogue cells (Table 1). Paclitaxel addition diminished the sensitivity of KB-15-PTX/099 to Am (Table 1 and Figure 9C). The paclitaxel-resistant A549-T12 cells that were grown in the presence of 2 nM paclitaxel during 72 h also did not exhibit resistance to Am: as shown in Table 1 and Figure 9D, IG_50_ for A549-T12 was not different from that of its sensitive analog A549, in contrast to KB-15-PTX/099 cells. These results demonstrate the importance of future experiments in the animal model.

Finally, to explore the Am effect on the non-human cells we used mouse melanoma B16 and mouse glioblastoma GL26 cell lines. As shown in Figure 9A and Figure S6, Am inhibited the proliferation of both lines, yielding IG_50_ comparable with that observed for example for MESSA and A549 cells (Table 1).

## Discussion

We show here that treatment with a semisynthetic drug amitozyn results in cell death by apoptosis. An Am treatment activated caspase-9, caspase-3 and PARP, common indicators of apoptosis and modulated the expression level of growth suppressor pRb. Indeed, after an Am treatment, we observed an increase in Rb and also in its phosphorylated forms. During the M-to-G1 transition, pRb is progressively dephosphorylated, returning to its growth-suppressive hypophosphorylated state [51], [52]. It appears thus that the Am treatment blocks cells in the M phase, as shown by the maximal level of hyperphosphorylated Rb observed after 12–24 hours post-treatment. However, after 48 hours of Am treatment, the level of phosphorylated Rb forms decreased as a result of partial M-to-G1 transition or apoptosis. The results of time-lapse (Figure S3) show, that exit M-G1 is not reductive and leads to the appearence of tetraploid cells.

Rb phosphorylation induced by Am treatment coincided with changes in the phosphorylation status of BubR1, the mitotic checkpoint kinase, suggesting changes in BubR1 activity. BubR1 protein mediates the proper attachment of microtubules to kinetochores and links the regulation of chromosome-spindle attachment to mitotic checkpoint signaling. Therefore, disruption of BubR1 activity results in chromosome instability and a loss of checkpoint control [53]. In vitro data suggest that the phosphorylation status of BubR1 is important for checkpoint inhibition of the anaphase-promoting complex/cyclosome [54] and for checkpoint-mediated mitotic arrest [55]. Our experiments showing prolonged phosphorylation of BubR1 protein after Am treatment clearly demonstrate the mitotic checkpoint activation. In addition, observed fluctations in the level Cyclin B1, a factor that controls the transition from G2 phase to M phase further suggests that Am induces the spindle checkpoint.

We observed that the Cyclin B1 amount that was first elevated decreased somewhat at a 48 h treatment (Figure 4). Cyclin B1 begins to accumulate in the G2 phase and reaches the peak level just before its destruction in metaphase. It begins to be degraded at the beginning of metaphase [56] by ubiquitin-mediated proteolysis [57]. Activating the spindle checkpoint by disrupting the spindle with nocodazole or colchicine inhibits degradation of cyclin B1 [58]. Similar results were obtained with an Am treatment; after 24 h of an Am treatment, the cyclin B1 level was stable, suggesting that Am induces the spindle checkpoint that inhibits cyclin B1 degradation.

The Bcl2 protein known as a marker of M-phase events [36] was phosphorylated upon an Am treatment, which suggests the appearance of mitosis-arrested cells [37]. Furthermore, Am treatment for 12–24 h significantly increased the level of phospho-PP1α (Thr 320) suggesting PP1 inhibition [38]. It is known that activated (dephosphorylated) PP1α dephosphorylates the phospho-Rb protein during late mitosis until complete dephosphorylation in the ensuing G1 period that suppresses the cell cycle progression. Treatment with Am for 48 h decreased the level of both phospho-PP1α (Thr 320) and phospho-Rb protein showing the partial transition of cells into the G1 phase while decreasing mitotic cell ratio. The sustained mitotic status of HeLa cells exposed to Am was supported also by the phosphorylation of histone H3, which is not detected in randomly cycling cells [40]. Phosphorylated histone H3 was diminished at an 48 h treatment with Am, demonstrating the decreasing mitotic cells ratio. Treatment of mitotic cells with inhibitor AZ 3146 but not with roscovitine decreased the level of cyclin B1, phosphorylated BubR1 and histone H3 (Figure 5A, B). AZ 3146, the mitotic checkpoint inhibitor, overrides the spindle checkpoint and promotes the mitotic exit.

It should be noted that the negative regulator of cell cycle such as p27 was affected upon an Am treatment. p27 was identified as a CDK (cyclin-dependent kinase) inhibitory protein induced by a variety of anti-proliferative signals that resulted in cells arrested in either G0 or G1 phase of the cell cycle [59]. *In vivo*, p27 can inhibit the kinase activities of a variety of cyclin-CDK complexes and is able to arrest cell cycle progression [60]. p27 extinction observed with Am treatment suggests that Am did not arrest cell cycle progression in the G1 phase. These data advocate for a strong disturbance of the cell cycle, leading to the accumulation of cells in the M phase. The mitotic block observed upon Am treatment is conceivably due to activation of the mitotic spindle assembly checkpoint.

Based on our results, we propose that Am-induced mitotic arrest and mitotic checkpoint can lead to three different scenarios. First – the cells stay blocked in mitosis by prolonged mitotic checkpoint that leads to the tetraploidization and passage of cells into the G1 phase of second round of cell division. Second – the cells with perturbed mitotic spindle remain capable of division, which results in aneuploidy with cells passing into the G1 phase of second round of cell division. Third – the cells arrested in mitosis undergo apoptosis that results in mitotic cell death. Each scenario might depend on the Am concentration and its ability to induce one or the other mitotic phenotype. Interestingly, significant cytotoxic effects induced by Am in HeLa cells were observed only after 48 h of treatment that coincided with maximal activation of caspase-3 and caspase-9 after mitotic checkpoint activation. These results show that Am predominantly kills actively dividing cells after their entry in mitosis.

Importantly, Am efficiently inhibited the growth of multidrug resistant cells such as MESSA Dx5 MDR that express hight level of active PgP pump and have the significant resistance for many cytostatics. Also, the paclitaxel-resistant cell line A549T12 was similarly inhibited by Am as its paclitaxel non-resistant counterpart, A549. Moreover, taxol-resistant KB15 PTX cells were even more sensitive to Am than taxol-sensitive KB cells. We suppose that the observed difference between A549T12 and KB 15 PTX sensitivity to Am depends on a particular tubulin mutation carried by these cells. A549T12 cells contain the heterozygous point mutation at alpha379 (Ser to Ser/Arg) in Kalpha1-tubulin [61]. Paclitaxel-resistant KB 15 PTX cells have a mutation Asp26Glu in the paclitaxel-binding region of beta-tubulin [49]. Comparison of anti-proliferative effect of Am on the HCT116 (p53 positive) with HCT116 (p53 null) cells demonstrated that cells with intact p53 were not more sensitive to Am than p53 null cells. Interestingly, the growth inhibition of HT29 cells with chromosomal instability exerted by Am was somewhat retarded when compared with the majority of studied cells. Published data show the modest anti-proliferative potency of chelidonine (Table S2). IG50 of chelidonine varies from 2.58 µM for A375 cells to over 141 µM for A549 cells [9], [62]–[66]. Calculated IG50 of chelidonine in Am for HeLa, HT29 and A549 cells was 0.478 µM, 1.7 µM, 1.45 µM respectively. These values are much lower than published IG50 of non-modified chelidonine: 85 µM, 16.7 µM and >141 µM for HeLa, HT29 and A549 cells respectively [63], [66].

We show here that Am increases the rate of tubulin polymerization *in vitro*, in the concentration-dependent fashion. Despite the activation of MTs, polymerization effect of Am is different from that of taxol, which increases the total polymer mass of tubulin and induces a completely different phenotype in HeLa cells. It is also different from that of the principal component of Am, chelidonine, which has been shown to inhibit the tubulin polymerization *in vitro* and to compete for the colchicine-binding site [7]. On the other side, both, Am and chelidonine, induce three similar types of aberrant mitotic phenotypes in HeLa cells, perturb the cell cycle, and arrest the cells in M phase. Thus, Am and chelidonine exert different effect on tubulin polymerization *in vitro* but induce similar mitotic aberrations in living cells. It is possible that the modification of chelidonine by ThioTEPA modulates the capacity of this alkaloid to inhibit the tubulin polymerization *in vitro*, rendering the modified drug, Am, a weak “stabilizator” of microtubule assembly *in vitro*. It is also conceivable that inside the cells amitozyn acquires depolymerization properties as a result of metabolic modification.

The celandine-derived semi synthetic drug Am can be prepared in a simple and rather inexpensive way. The dose-dependent and reversible anti-proliferative effect of Am observed in several transformed cell lines opens the way to further preclinical evaluation of this cytostatic drug.

## Supporting Information

Figure S1 **HPLC-UV of amitozyn performed at 280 nm wavelength.**
(TIF)

Figure S2 **MS/MS fragmentation of chelidonine. (**A) Chelidonine standard (B) Chelidonine in Am, t_R_ = 12.67. (C) Chelidonine in Am, t_R_ = 16.19
(TIF)

Figure S3 **Antiproliferative effect of amitozyn. (**A) Time-lapse visualisation of HeLa H2B-GFP cells treated with Am. Non-synchronized cells were exposed with 125 µg/ml Am and filmed with an inverted microscope as described in Materials and methods. Differential interference contrast (DIC) and GFP uorescence were monitored every 10 min for 72 h. The arrows show interphase (I), mitotic (M), micronucleated (MN) and apoptotic (A) cells respectively. (B) Statistical analysis of mitotic death and micronucleation upon Am treatment. Portion of 150 cells in mitosis were tested and percentage of mitotic death (MD) and micronucleated (MN) cells was assessed.
(TIF)

Figure S4 **(A) Analysis of distance between spindle poles in control and Am treated cells.** (B) Comparison of mitotic phenotypes observed upon treatment with 10 nM vinblastine (Vin), 10 nM paclitaxel (Ptx), 1 µg/ml nocodazole (N) and 50 nM colchicine (Col).
(TIF)

Figure S5 **Effect of chelidonine on tubulin polymerization** ***in vitro*** **.** Tubulin (60 µM) was polymerized for 20 min at 37°C in the presence of 0–10 µM chelidonine as described in Materials and methods .
(TIF)

Figure S6 **Effect of amitozyn on murine cells.** B16 (A) and GL26 (B) cells were exposed to different Am concentrations for up to 72 h and analyzed by FACScan.
(TIF)

Table S1 **Amitozyn composition.**
(XLS)

Table S2 **Antiproliferative potential of chelidonine (literature data).**
(XLS)

## References

[1] Widmann H (1955) Effect of celandine alkaloids on the growth of mouse ascites tumor. Arch Geschwulstforsch 9: 6–32. 13327901

[2] Aminev AM (1963) The addition to the treatment of benign polyposis with Herba chelidonii. Am J Proctol 14: 25–27. 14012603

[3] Savchak VI (1976) Case of facial pepillomatosis successfully treated with celandine juice. Vestn Dermatol Venerol 3: 77. 961056

[4] Sarkozi A, Janicsak G, Kursinszki L, Kery A (2006) Alkaloid Composition of Chelidonium majus L. Studied by Different Chromatographic Techniques. Chromatographia 63: 81–86.

[5] Lettre H, Albrecht M (1942) Narcotin, ein Mitosegift. Natuwiss 30: 184–185.

[6] Joubert A, Lottering ML, Panzer A (2004) Influence of chelidonine, an inhibitor of tubulin polymerisation on tyrosine kinase activity in normal, transformed and malignant cell lines. Biomedical Research 25: 27–33.

[7] Wolff J, Knipling L (1993) Antimicrotubule properties of benzophenanthridine alkaloids. Biochemistry 32: 13334–13339. 7902132 10.1021/bi00211a047

[8] Panzer A, Joubert AM, Bianchi PC, Hamel E, Seegers JC (2001) The effects of chelidonine on tubulin polymerisation, cell cycle progression and selected signal transmission pathways. Eur J Cell Biol 80: 111–118. 11211931 10.1078/0171-9335-00135

[9] Kaminskyy V, Kulachkovskyy O, Stoika R (2008) A decisive role of mitochondria in defining rate and intensity of apoptosis induction by different alkaloids. Toxicol Lett 177: 168–181. 18325696 10.1016/j.toxlet.2008.01.009

[10] Kaminskyy V, Lin KW, Filyak Y, Stoika R (2008) Differential effect of sanguinarine, chelerythrine and chelidonine on DNA damage and cell viability in primary mouse spleen cells and mouse leukemic cells. Cell Biol Int 32: 271–277. 18029203 10.1016/j.cellbi.2007.09.004

[11] Caballero-George C, Vanderheyden PM, Apers S, Van den Heuvel H, Solis PN, et al (2002) Inhibitory activity on binding of specific ligands to the human angiotensin II AT(1) and endothelin 1 ET(A) receptors: bioactive benzo(c)phenanthridine alkaloids from the root of Bocconiafrutescens. Planta Med 68: 770–775. 12357384 10.1055/s-2002-34406

[12] Vavrecková C, Gawlik I, Müller K (1996) Benzophenanthridine alkaloids of Chelidonium majus; I. Inhibition of 5- and 12-lipoxygenase by a non-redox mechanism. Planta Med 62: 397–401. 9005450 10.1055/s-2006-957924

[13] Drsata J, Ulrichov J, Walterov D (1996) Sanguinarine and Chelerythrine as Inhibitors of Aromatic Amino Acid Decarboxylase. J Enzyme Inhib Med Chem 10: 231–237. 10.3109/14756369609036530 8872743

[14] Lopus M, Panda D (2006) Thebenzophenanthridine alkaloid sanguinarine perturbs microtubule assembly dynamics through tubulin binding. A possible mechanism for its antiproliferative activity. FEBS J 273: 2139–2150. 16649991 10.1111/j.1742-4658.2006.05227.x

[15] Lee SS, Kai M, Lee MK (2001) Inhibitory effects of sanguinarine on monoamine oxidase activity in mouse brain. Phytotherapy Research 15: 167–169. 11268121 10.1002/ptr.703

[16] Seifen E, Adams RJ, Riemer RK (1979) Sanguinarine: a positive inotropic alkaloid which inhibits cardiac Na+, K+ ATPase. Eur J Pharmacol 60: 373–377. 230984 10.1016/0014-2999(79)90245-0

[17] Herbert JM, Augereau JM, Gleye J, Maffrand JP (1990) Chelerythrine is a potent and specific inhibitor of protein kinase C. Biochem Biophys Res Commun. 172: 993–999. 10.1016/0006-291x(90)91544-3 2244923

[18] Lee SK, Qing WG, Mar W, Luyengi L, Mehta RG, et al (1998) Angoline and chelerythrine, benzophenanthridine alkaloids that do not inhibit protein kinase C. J Biol Chem. 273: 19829–19833. 10.1074/jbc.273.31.19829 9677417

[19] Matkar SS, Wrischnik LA, Hellmann-Blumberg U (2008) Sanguinarine causes DNA damage and p53-independent cell death in human colon cancer cell lines. Chem Biol Interact 10: 63–71. 10.1016/j.cbi.2007.12.006 18243168

[20] Bai LP, Zhao ZZ, Cai Z, Jiang ZH (2006) DNA-binding affinities and sequence selectivity of quaternary benzophenanthridine alkaloids sanguinarine, chelerythrine, and nitidine. Bioorg Med Chem 15: 5439–5445. 10.1016/j.bmc.2006.05.012 16730995

[21] Maiti M, Nandi R, Chaudhuri K (1982) Sanguinarine: a monofunctional intercalating alkaloid. FEBS Lett 7: 280–284. 10.1016/0014-5793(82)80152-x 7106291

[22] Tanabe H, Suzuki H, Nagatsu A, Mizukami H, Ogihara Y, et al (2006) Selective inhibition of vascular smooth muscle cell proliferation by coptisine isolated from Coptis rhizoma, one of the crude drugs composing Kampo medicines Unsei-in. Phytomedicine 13: 334–342. 16635741 10.1016/j.phymed.2005.02.001

[23] Colombo ML, Bugatti C, Mossa A (2001) Cytotoxicity evaluation of natural coptisine and synthesis of coptisine from berberine. Farmaco 56: 403–409. 11482767 10.1016/s0014-827x(01)01121-1

[24] Tanaka T, Metori K, Mineo S, Hirotani M, Furuya T, et al (1993) Inhibitory effects of berberine-type alkaloids on elastase. Planta Med 59: 200–202. 8316586 10.1055/s-2006-959651

[25] Fil’chenkov OO, Zavelevych MP, Khranovs’ka NM, Zaïka LA, Potopal’s’ky AI (2006) Modified alkaloids from Chelidonium majus L. induce G2/M arrest, caspase-3 a ctivation, and apoptosis in human acute lymphoblastic leukemia MT-4 cells. Ukr Biokhim Zh 78: 81–87. 17290785

[26] Tcherniuk S, Skoufias DA, Labriere C, Rath O, Gueritte F (2010) Relocation of Aurora B and surviving from centromeres to the central spindle impaired by a kinesin-specific MKLP-2 inhibitor. Angew Chem Int Ed Engl 49: 8228–8231. 20857469 10.1002/anie.201003254

[27] Tcherniuk S, Deshayes S, Sarli V, Divita G, Abrieu A (2011) UA62784 is a cytotoxic inhibitor of microtubules, not CENP-E. Chem Biol 18: 631–641. 21609844 10.1016/j.chembiol.2011.03.006

[28] De Azevedo WF, Leclerc S, Meijer L, Havlicek L, Strnad M, et al (1997) Inhibition of cyclin-dependent kinases by purine analogues: crystal structure of human cdk2 complexed with roscovitine. Eur J Biochem 243: 518–526. 9030780 10.1111/j.1432-1033.1997.0518a.x

[29] Hewitt L, Tighe A, Santaguida S, White AM, Jones CD, et al (2010) Sustained Mps1 activity is required in mitosis to recruit O-Mad2 to the Mad1-C-Mad2 core complex. J Cell Biol 190: 25–34. 20624899 10.1083/jcb.201002133 PMC2911659

[30] Robley C, Williams JR, Lee JC (1982) Preparation of Tubulin from Brain. In: Frederiksen DW, Cunningham LW, editors. Structural and Contractile Proteins Part B: The Contractile Apparatus and the Cytoskeleton. Methods Enzymol 85, Elsevier Science. pp.376–385. 10.1016/0076-6879(82)85038-6 7121276

[31] Davis FM, Tsao TY, Fowler SK, Rao PN (1983) Monoclonal antibodies to mitotic cells. Proc Natl Acad Sci USA 80: 2926–2930. 6574461 10.1073/pnas.80.10.2926 PMC393946

[32] Fernandes-Alnemri T, Litwack G, Alnemri ES (1994) CPP32, a novel human apoptotic protein with homology to Caenorhabditiselegans cell death protein Ced-3 and mammalian interleukin-1 beta-converting enzyme. J Biol Chem 269: 30761–30764. 7983002

[33] Tewari M, Quan LT, O’Rourke K, Desnoyers S, Zeng Z, et al (1995) Yama/CPP32 beta, a mammalian homolog of CED-3, is a CrmA-inhibitable protease that cleaves the death substrate poly(ADP-ribose) polymerase. Cell 81: 801–809. 7774019 10.1016/0092-8674(95)90541-3

[34] Li P, Nijhawan D, Budihardjo I, Srinivasula SM, Ahmad M, et al (1997) Cytochrome c and dATP-dependent formation of Apaf-1/caspase-9 complex initiates an apoptotic protease cascade. Cell 91: 479–489. 9390557 10.1016/s0092-8674(00)80434-1

[35] Li W, Lan Z, Wu H, Wu S, Meadows J, et al (1999) BubR1 Phosphorylation Is Regulated during Mitotic Checkpoint Activation. Cell Growth Differ 10: 769–775. 10593653

[36] Ling YH, Tornos C, Perez-Soler R (1998) Phosphorylation of Bcl-2 is a marker of M phase events and not a determinant of apoptosis. J Biol Chem 273: 18984–18991. 9668078 10.1074/jbc.273.30.18984

[37] Chadebech P, Brichese L, Baldin V, Vidal S, Valette A (1999) Phosphorylation and proteasome-dependent degradation of Bcl-2 in mitotic-arrested cells after microtubule damage. Biochem Biophys Res Commun 262: 823–827. 10471409 10.1006/bbrc.1999.1291

[38] Dohadwala M, da Cruz e Silva EF, Hall FL, Williams RT, Carbonaro-Hall DA, et al (1994) Phosphorylation and inactivation of protein phosphatase 1 by cyclin-dependent kinases. Proc Natl Acad Sci USA 91: 6408–6412. 8022797 10.1073/pnas.91.14.6408 PMC44211

[39] Kwon YG, Lee SY, Choi Y, Greengard P, Nairn AC (1997) Cell cycle-dependent phosphorylation of mammalian protein phosphatase 1 by cdc2 kinase. Proc Natl Acad Sci USA 94: 2168–2173. 9122166 10.1073/pnas.94.6.2168 PMC20059

[40] Hendzel MJ, Wei Y, Mancini MA, Van Hooser A, Ranalli T, et al (1997) Mitosis-specific phosphorylation of histonee H3 initiates primarily within pericentromeric heterochromatin during G2 and spreads in an ordered fashion coincident with mitotic chromosome condensation. Chromosoma 106: 348–360. 9362543 10.1007/s004120050256

[41] Ross W, Rowe T, Glisson B, Yalowich J, Liu L (1984) Role of topoisomerase II in mediating epipodophyllotoxin-induced DNA cleavage. Cancer Res 44: 5857–5860. 6094001

[42] Haslam G, Wyatt D, Kitos PA (2000) Estimating the number of viable animal cells in multi-well cultures based on their lactate dehydrogenase activities. Cytotechnology 32: 63–75. 19002967 10.1023/A:1008121125755 PMC3449446

[43] Cahill DP, Lengauer C, Yu J, Riggins GJ, James KV, et al (1998) Mutations of mitotic checkpoint genes in human cancers. Nature 392: 300–303. 9521327 10.1038/32688

[44] Galmarini CM, Kamath K, Vanier-Viornery A, Hervieu V, Peiller E, et al (2003) Drug resistance associated with loss of p53 involves extensive alterations in microtubule composition and dynamics. BJC 88: 1793–1799. 12771997 10.1038/sj.bjc.6600960 PMC2377136

[45] Gasco M, Crook T (2003) p53 family members and chemoresistance in cancer: what we know and what we need to know. Drug Resist Updat 6: 323–328. 14744496 10.1016/j.drup.2003.11.001

[46] Nakanishia Y, Kawasakia M, Baia F, Takayamaa K, Peia XH (1999) Expression of p53 and Glutathione S-Transferase- Relates to Clinical Drug Resistance in Non-Small Cell Lung Cancer. Oncology 57: 318–323. 10575319 10.1159/000012068

[47] Vasey PA, Jones NA, Jenkins S, Dive C, Brown R (1996) Cisplatin, camptothecin, and taxol sensitivities of cells with p53 - associated multidrug resistance. Mol Pharmacol 50: 1536–1540. 8967975

[48] Harker WG, Sikic BI (1985) Multidrug (Pleiotropic) Resistance in Doxorubicin-selected Variants of the Human Sarcoma Cell Line MES-SA. Cancer Res 45: 4091–4096. 4028002

[49] Hari M, Loganzo F, Annable T, Tan X, Musto S, et al (2006) Paclitaxel-resistant cells have a mutation in the paclitaxel-binding region of beta-tubulin (Asp26Glu) and less stable microtubules. Mol Cancer Ther 5: 270–278. 16505100 10.1158/1535-7163.MCT-05-0190

[50] Kavallaris M, Kuo DY, Burkhart CA, Regl DL, Norris MD, et al (1997) Taxol-resistant epithelial ovarian tumors are associated with altered expression of specific beta-tubulin isotypes. J Clin Invest 100: 1282–1293. 9276747 10.1172/JCI119642 PMC508306

[51] Nelson DA, Krucher NA, Ludlow JW (1997) High molecular weight protein phosphatase type 1 dephosphorylates the retinoblastoma protein. J Biol Chem 272: 4528–4535. 9020179 10.1074/jbc.272.7.4528

[52] Ludlow JW, Glendening CL, Livingston DM, De Carprio JA (1993) Specific enzymatic dephosphorylation of the retinoblastoma protein. Mol Cell Biol 13: 367–372. 8380224 10.1128/mcb.13.1.367-372.1993 PMC358916

[53] Shin HJ, Baek KH, Jeon AH, Park MT, Lee SJ, et al (2003) Dual roles of human BubR1, a mitotic checkpoint kinase, in the monitoring of chromosomal instability. Cancer Cell 4: 483–497. 14706340 10.1016/s1535-6108(03)00302-7

[54] Huang H, Hittle J, Zappacosta F, Annan RS, Hershko A, et al (2008) Phosphorylation sites in BubR1 that regulate kinetochore attachment, tension, and mitotic exit. J Cell Biol 183: 667–680. 19015317 10.1083/jcb.200805163 PMC2582891

[55] Wong OK, Fang G (2007) Cdk1 phosphorylation of BubR1 controls spindle checkpoint arrest and Plk1-mediated formation of the 3F3/2 epitope. J Cell Biol 19: 611–617. 10.1083/jcb.200708044 PMC2080899 17998400

[56] Clute P, Pines J (1999) Temporal and spatial control of cyclin B1 destruction in metaphase. Nat Cell Biol 1: 82–87. 10559878 10.1038/10049

[57] Glotzer M, Murray AW, Kirschner MW (1991) Cyclin is degraded by the ubiquitin pathway. Nature 349: 132–138. 1846030 10.1038/349132a0

[58] Whitfield WGF, Gonzalez C, Maldonado-Codina G, Glover DM (1990) The A- and B-type cyclins of Drosophila are accumulated and destroyed in temporally distinct events that define separable phases of the G2-M transition. EMBO J 9: 2563–2572. 2142452 10.1002/j.1460-2075.1990.tb07437.x PMC552287

[59] Nourse J, Firpo E, Flanagan WM, Coats S, Polyak K, et al (1994) Roberts, lnterleukin-2-mediated elimination of the p27Kipl cyclin-dependent kinase inhibitor prevented by rapamycin. Nature 372: 570–573. 7990932 10.1038/372570a0

[60] Toyoshima H, Hunter T (1994) p27, a novel inhibitor of G1 cyclin-Cdk protein kinase activity, is related to p21. Cell 78: 67–74. 8033213 10.1016/0092-8674(94)90573-8

[61] Martello LA, Verdier-Pinard P, Shen HJ, He L, Torres K, et al (2003) Elevated levels of microtubule destabilizing factors in a Taxol-resistant/dependent A549 cell line with an alpha-tubulin mutation. Cancer Res 63: 1207–1213. 12649178

[62] Park JE, Cuong TD, Hung TM, Lee I, Na M, et al (2011) Alkaloids from Chelidonium majus and their inhibitory effects on LPS-induced NO production in RAW264.7 cells. Bioorg Med Chem Lett 21: 6960–6963. 22024033 10.1016/j.bmcl.2011.09.128

[63] Paul A, Bishayee K, Ghosh S, Mukherjee A, Sikdar S, et al (2012) Chelidonine isolated from ethanolic extract of Chelidonium majus promotes apoptosis in HeLa cells through p38-p53 and PI3K/AKT signalling pathways. J Chin Integr Med/Zhong Xi Yi Jie He Xue Bao 10: 1025–1038. 10.3736/jcim20120912 22979935

[64] Hammerová J, Uldrijan S, Táborská E, Slaninová I (2011) Benzo[c]phenanthridine alkaloids exhibit strong anti-proliferative activity in malignant melanoma cells regardless of their p53 status. J Dermatol Sci 62: 22–35. 21324654 10.1016/j.jdermsci.2011.01.006

[65] Kemény-Beke A, Aradi J, Damjanovich J, Beck Z, Facskó A, et al (2006) Apoptotic response of uveal melanoma cells upon treatment with chelidonine, sanguinarine and chelerythrine. Cancer Lett 237: 67–75. 16019128 10.1016/j.canlet.2005.05.037

[66] Lee J, Shon MY, Jang DS, Ha TJ, Hwang SW, et al (2005) Cytotoxic Isoquinoline Alkaloids from Chelidonium majus var. asiaticum. Agric Chem Biotechnol 48: 198–201.

