# Correction: Amitozyn Impairs Chromosome Segregation and Induces Apoptosis via Mitotic Checkpoint Activation

**DOI:** 10.1371/annotation/0b7ab40b-2246-4426-a98d-36b028029c5c

**Published:** 2013-06-27

**Authors:** Bastien Herman, Aldrian Gudrun, Anatoly I. Potopalsky, Jadwiga Chroboczek, Sergey O. Tcherniuk

The name of the first author was spelled incorrectly. The correct name is: Bastien Hermant. 

The correct citation is: Hermant B, Gudrun A, Potopalsky AI, Chroboczek J, Tcherniuk SO (2013) Amitozyn Impairs Chromosome Segregation and Induces Apoptosis via Mitotic Checkpoint Activation. PLoS ONE 8(3): e57461. doi:10.1371/journal.pone.0057461. 

