# Correction: Amitozyn Impairs Chromosome Segregation and Induces Apoptosis via Mitotic Checkpoint Activation

**DOI:** 10.1371/journal.pone.0118039

**Published:** 2015-02-03

**Authors:** The PLOS ONE Staff

The structural formula of alkaloid chelidonine in Fig. 1 is incorrect. Please view the corrected Fig. 1 here.

**Figure 1:**
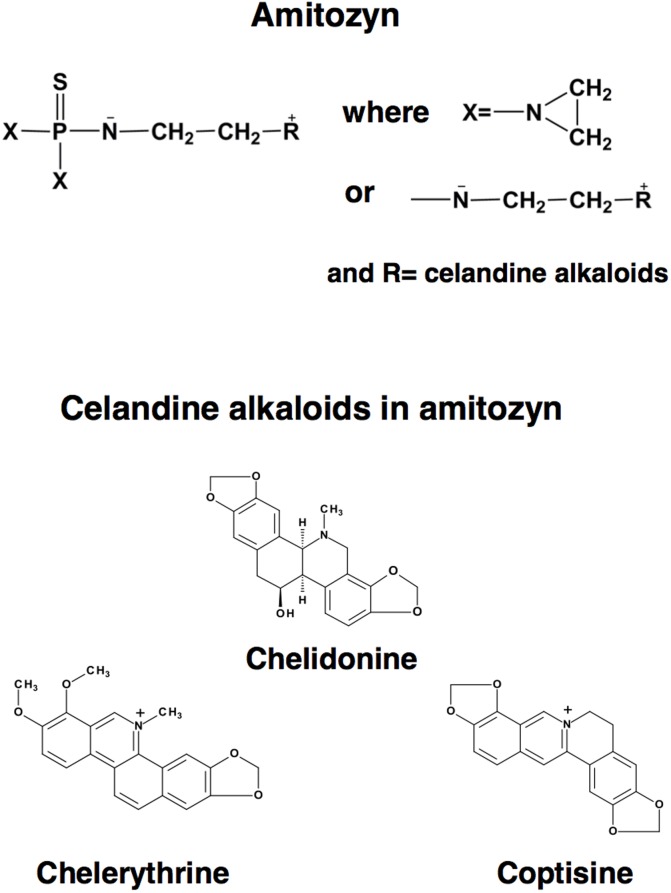
Structure of amitozyn and celandine alkaloids.
