# Supplementary figures and images for: Amitozyn Impairs Chromosome Segregation and Induces Apoptosis via Mitotic Checkpoint Activation
Source: PLoS One. 2013 Mar 7;8(3):e57461. doi: 10.1371/journal.pone.0057461 (PMC3591406; doi:10.1371/journal.pone.0057461)

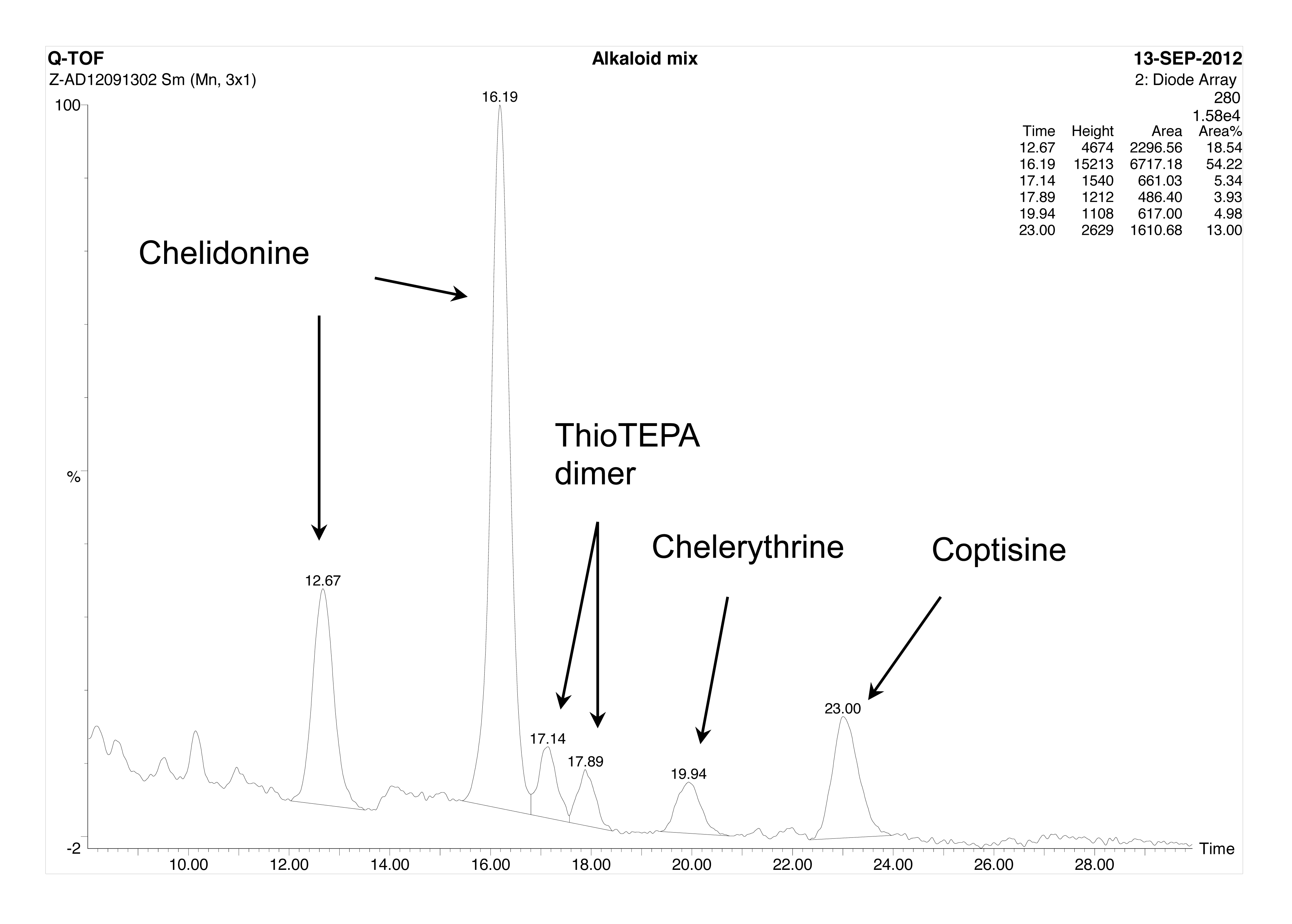

Supplement: Figure S1 — HPLC-UV of amitozyn performed at 280 nm wavelength. (TIF) [file pone.0057461.s001.tif]

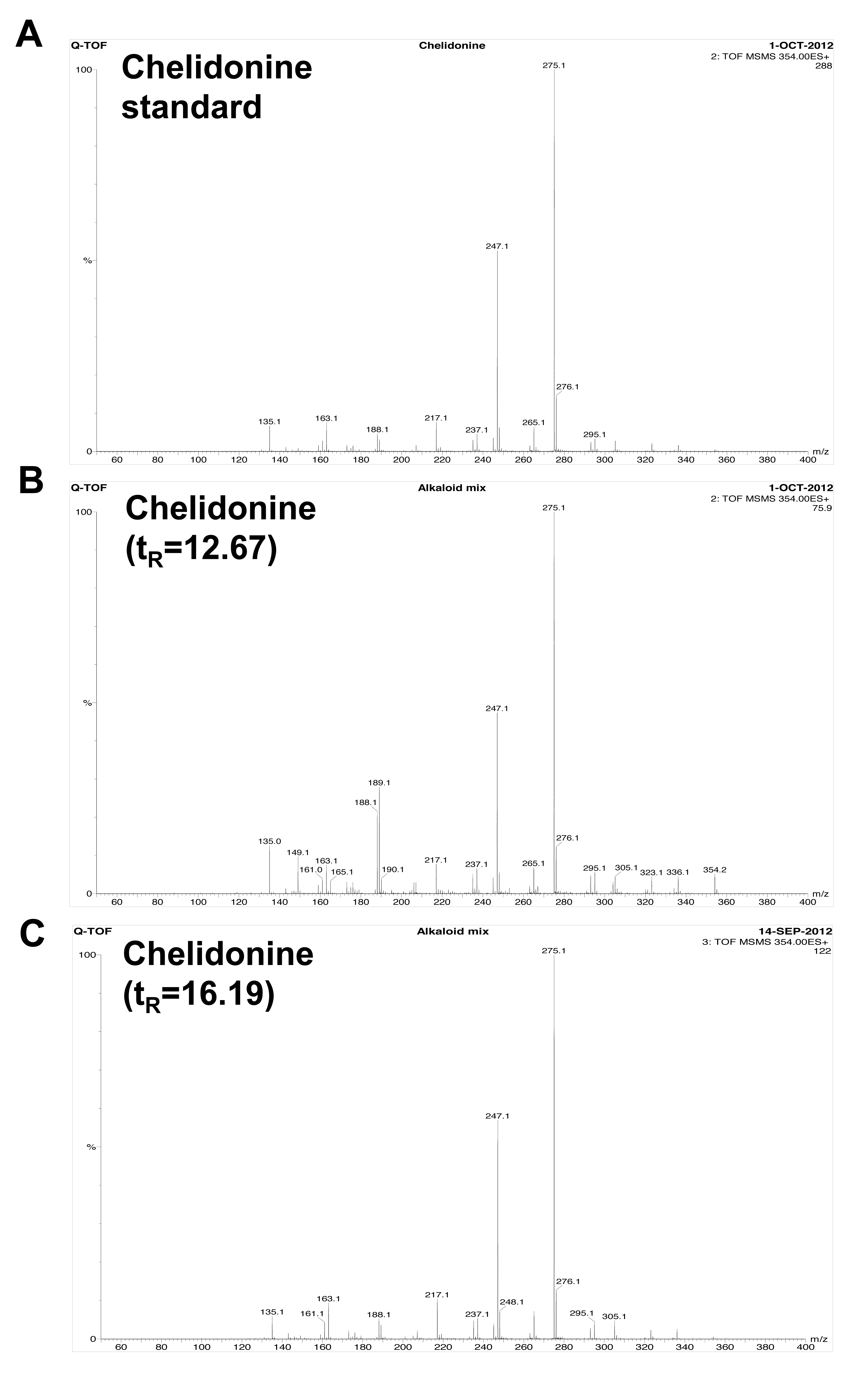

Supplement: Figure S2 — MS/MS fragmentation of chelidonine. (A) Chelidonine standard (B) Chelidonine in Am, tR = 12.67. (C) Chelidonine in Am, tR = 16.19 (TIF) [file pone.0057461.s002.tif]

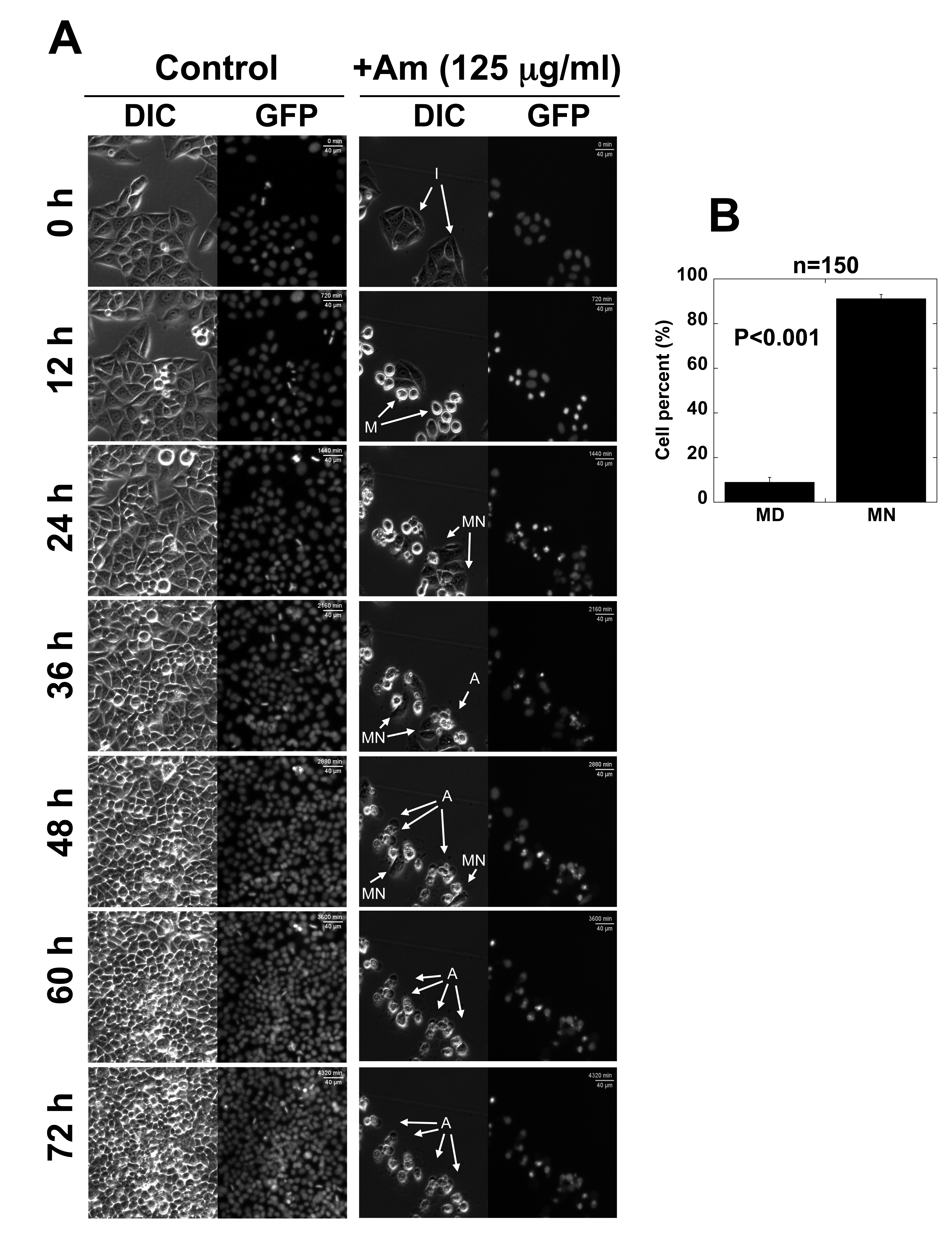

Supplement: Figure S3 — Antiproliferative effect of amitozyn. (A) Time-lapse visualisation of HeLa H2B-GFP cells treated with Am. Non-synchronized cells were exposed with 125 µg/ml Am and filmed with an inverted microscope as described in Materials and methods. Differential interference contrast (DIC) and GFP uorescence were monitored every 10 min for 72 h. The arrows show interphase (I), mitotic (M), micronucleated (MN) and apoptotic (A) cells respectively. (B) Statistical analysis of mitotic death and micronucleation upon Am treatment. Portion of 150 cells in mitosis were tested and percentage of mitotic death (MD) and micronucleated (MN) cells was assessed. (TIF) [file pone.0057461.s003.tif]

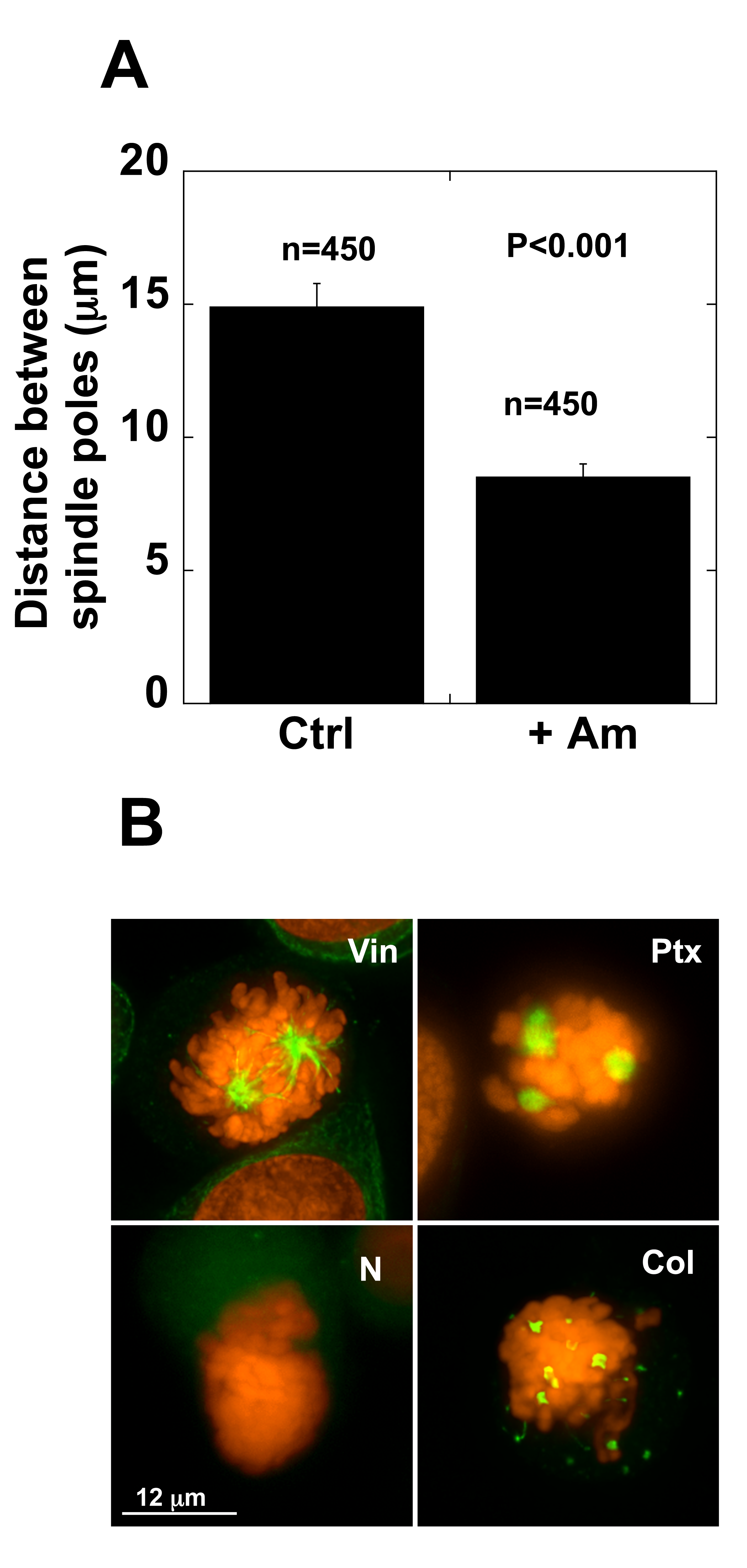

Supplement: Figure S4 — (A) Analysis of distance between spindle poles in control and Am treated cells. (B) Comparison of mitotic phenotypes observed upon treatment with 10 nM vinblastine (Vin), 10 nM paclitaxel (Ptx), 1 µg/ml nocodazole (N) and 50 nM colchicine (Col). (TIF) [file pone.0057461.s004.tif]

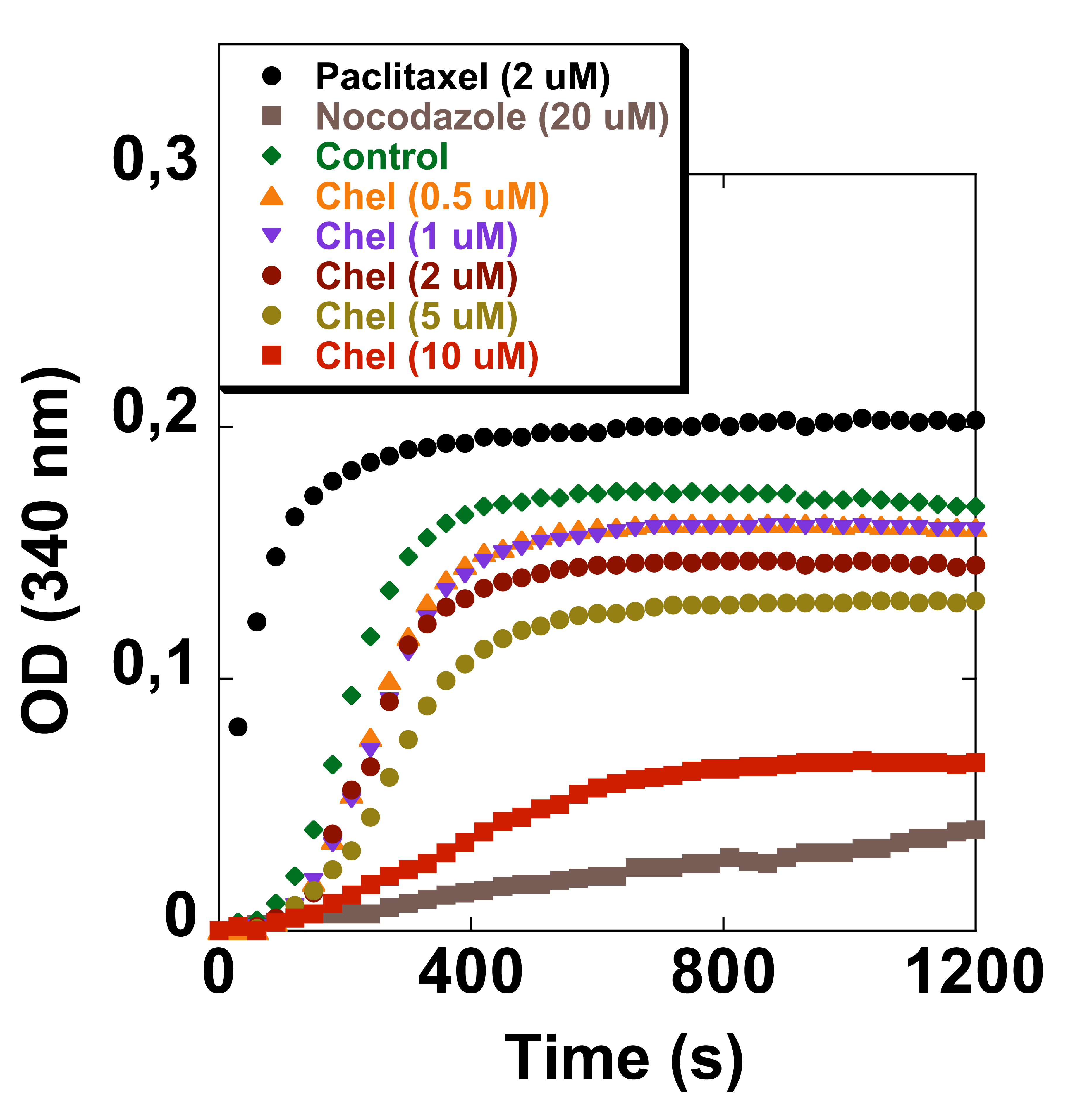

Supplement: Figure S5 — Effect of chelidonine on tubulin polymerization in vitro . Tubulin (60 µM) was polymerized for 20 min at 37°C in the presence of 0–10 µM chelidonine as described in Materials and methods . (TIF) [file pone.0057461.s005.tif]

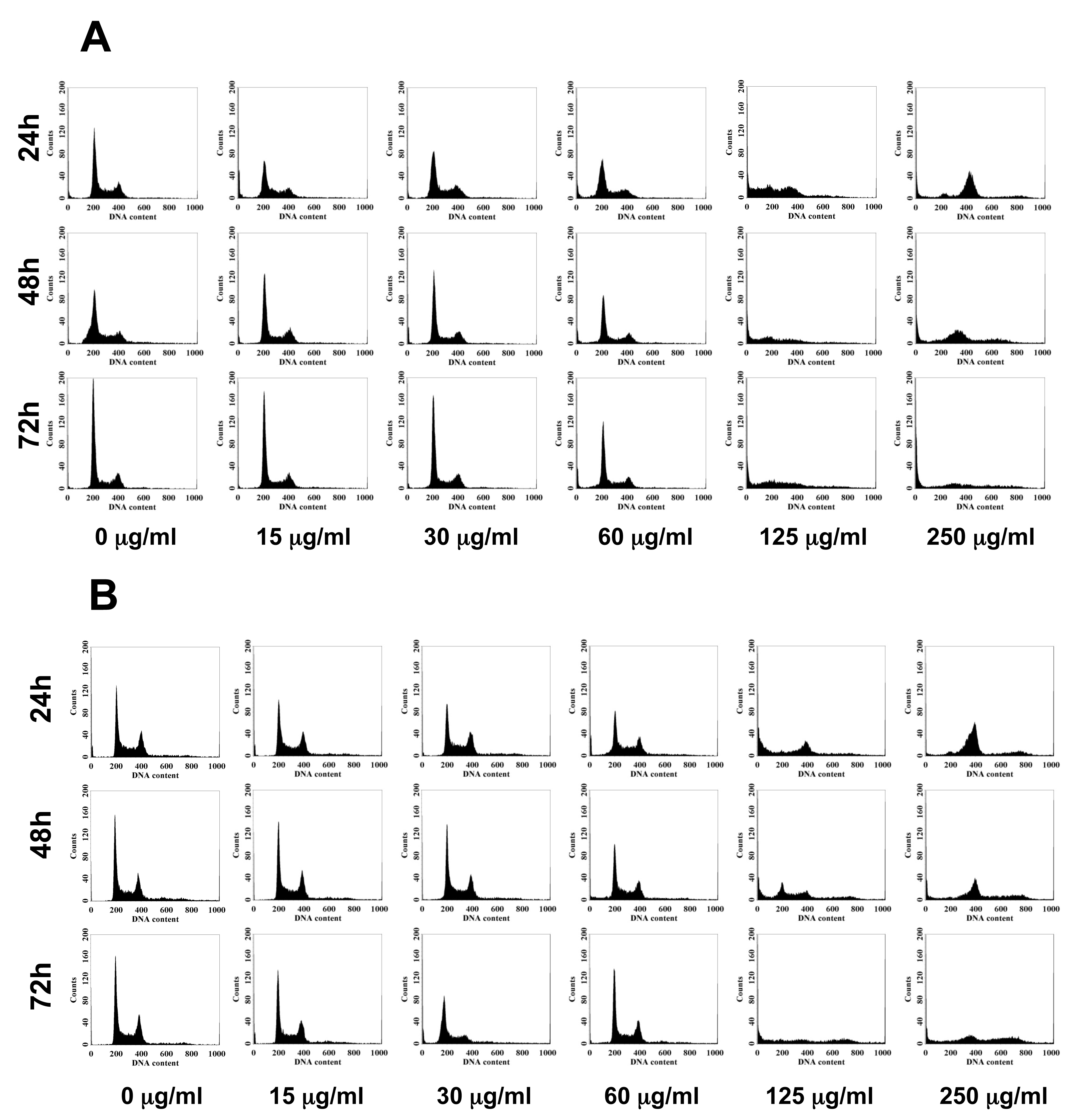

Supplement: Figure S6 — Effect of amitozyn on murine cells. B16 (A) and GL26 (B) cells were exposed to different Am concentrations for up to 72 h and analyzed by FACScan. (TIF) [file pone.0057461.s006.tif]
